# Synthesis, Structural Characterization, and In Silico Evaluation of the Salicylidene Schiff Base 4‑{(*E*)‑[(2,3-Dihydroxyphenyl)methylidene]amino}-2-hydroxybenzoic Acid as a Promising Scaffold for Human Transthyretin Inhibitor

**DOI:** 10.1021/acsomega.5c07247

**Published:** 2026-01-21

**Authors:** Patryk Nowak, Artur Sikorski

**Affiliations:** Faculty of Chemistry, University of Gdańsk, W. Stwosza 63, Gdańsk 80-308, Poland

## Abstract

The Schiff base derived from the condensation of 4-aminosalicylic acid with 2,3-dihydroxybenzaldehyde was synthesized and characterized by using experimental techniques, theoretical calculations, and in silico methods. Single-crystal X-ray diffraction analysis showed that the compound crystallizes in the triclinic *P*1̅ space group with two molecules in the asymmetric unit, both adopting a zwitterionic keto form. In silico studies predict high gastrointestinal absorption, low toxicity, and potential activity as a transthyretin (TTR) inhibitor, with binding affinity comparable to that of the drug Tolcapone, suggesting that the synthesized compound may be a promising scaffold for a TTR stabilizer.

## Graphical abstract

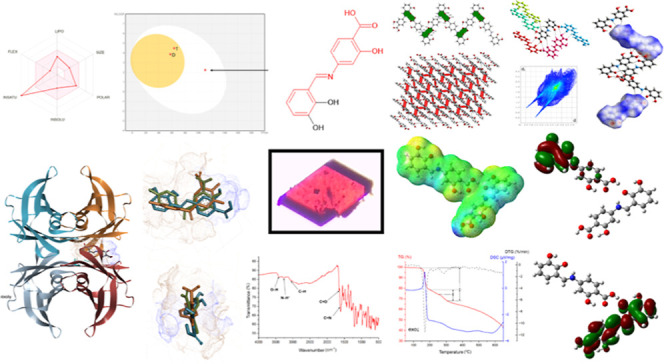

## Introduction

Advancements in computational methods have revolutionized the assessment of in silico properties, enabling faster and more cost-effective drug development.^1–3^ When combined with experimental data, in silico models improve the accuracy of predictions regarding the absorption, distribution, metabolism, excretion, and toxicity (ADMET) of small molecules as well as their interactions with biologically relevant macromolecules. The continuous refinement of machine learning algorithms and the expansion of high-quality experimental data sets further enhance the reliability of these tools. Consequently, researchers can prioritize promising drug candidates earlier in the development process, reducing late-stage failures and minimizing the risks associated with adverse effects. Thus, integrating in silico methods like virtual screening, ADMET profiling, and molecular docking is crucial in drug discovery.^4–6^

From the point of view of designing new drugs, a promising group of compounds is Schiff bases. This group of compounds has demonstrated a wide range of therapeutic potential, including antimicrobial,^7,8^ antitumor,^9,10^ antioxidant,^11,12^ and anti-inflammatory activities, among others.^13,14^ They have been also found to inhibit various enzymes, such as acetylcholinesterase, butyrylcholinesterase, carbonic anhydrase, urease, or monoamine oxidase.^15–21^

Taking into account, we focused our attention on salicylidene Schiff bases.^22^ These compounds are formed by the condensation of aminobenzoic acids with salicylaldehyde derivatives, both of which exhibit medicinal properties. In particular, salicylic acid derivatives such as acetylsalicylic acid, methyl salicylate, salicylamide, and 5-aminosalicylic acid are widely used in the treatment of various diseases.^23,24^ Given their therapeutic potential, we focused on a particularly promising class of salicylic acid derivatives: aminosalicylic acids. Among them, 4-aminosalicylic acid serves as a second-line drug for the treatment of multidrug-resistant tuberculosis.^25,26^ Although initially considered a first-line treatment, it was replaced by ethambutol in the early 1960s.^27^ Furthermore, 4-aminosalicylic acid has demonstrated effectiveness in the treatment of Crohn’s disease,^28^ ulcerative colitis,^29^ and inflammatory bowel disease.^30^

In turn, salicylaldehyde derivatives, especially dihydroxybenzaldehydes, represent an interesting subclass of polyhydroxyphenol derivatives.^31,32^ Polyhydroxyphenolic compounds constitute a diverse class of molecules with a broad spectrum of biological activities, including antioxidant, antitumor, antiseptic, and enzyme-inhibitory properties.^33–35^ Among them, 2,3-dihydroxybenzaldehyde has been reported to exhibit antimicrobial activity.^36^

Based on the previously discussed properties of aminosalicylic acids and polyphenolic aldehydes, we planned the synthesis of Schiff bases derived from various aminosalicylic acids and 2,3-dihydroxybenzaldehyde. Although the synthesis of these compounds has been reported,^37,38^ their crystal structures have not been reported due to the lack of suitable single crystals. However, we successfully obtained one of these Schiff bases in the crystalline form, resulting from the condensation of 4-aminosalicylic acid with 2,3-dihydroxybenzaldehyde. This compound exhibits a certain degree of visual structural similarity to known Transthyretin (TTR) stabilizers such as Tafamidis, Diflunisal, and Tolcapone,^39^ as presented in Scheme 1.

**Scheme 1:**
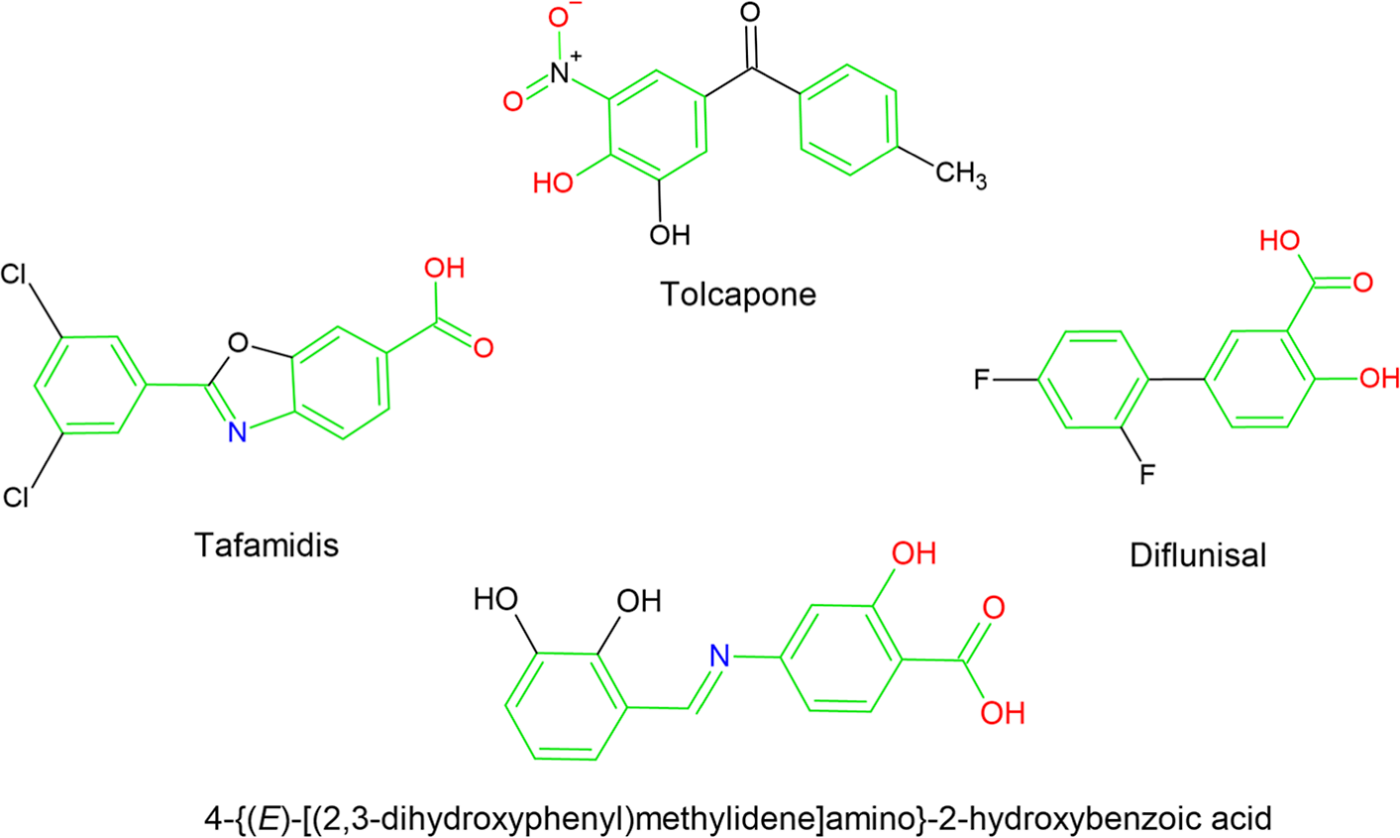
Molecular Structures of Selected TTR Stabilizers and Schiff Base Derived from 4-Aminosalicylic Acid and 2,3-Dihydroxybenzaldehyde (the Similar Structural Fragments Are Colored)

TTR is a tetrameric transport protein responsible for distributing thyroxine and retinol-binding protein in plasma and cerebrospinal fluid.^40^ Its wild-type form is intrinsically amyloidogenic, leading to amyloid fibril formation in elderly individuals and contributing to senile systemic amyloidosis.^41,42^ Tetrameric stability plays a crucial role in modulating TTR’s interaction with the Aβ peptide, preventing its aggregation and associated toxicity.^43–45^ Consequently, stabilizing the TTR tetramer has been recognized as a key therapeutic strategy for familial amyloid polyneuropathy.^46–48^ This stabilization can be achieved using small molecules structurally similar to thyroxine, which bind within the central TTR binding channel and prevent early tetramer dissociation.^49,50^ Identifying such stabilizing compounds remains a major focus in the development of therapeutic approaches for TTR-related amyloidosis.^51–63^ This is supported by crystallographic studies, which have reported 240 structures of human TTR complexed with over 100 small ligands including Tafamidis, Tolcapone, and Diflunisal.^64–66^

Herein, we present the results of the synthesis and crystallization of 4-{(*E*)-[(2,3-dihydroxyphenyl)­methylidene]­amino}-2-hydroxybenzoic acid, together with its spectroscopic and structural characterization, including a detailed analysis of intermolecular interactions occurring in the crystal structure. Additionally, in silico ADMET prediction and molecular docking studies were performed to evaluate their potential as a therapeutic agent.

## Experimental Section

### Materials

Reagents from Sigma-Aldrich were used without further purification. Purity was verified by melting point measurements (Büchi M-545) and crystal images were captured using a Discovery Femto Polar Digital Microscope with a 3 MP camera.

### Synthesis of 4-{(*E*)-[(2,3-Dihydroxyphenyl)­methylidene]­amino}-2-hydroxybenzoic Acid

4-Aminosalicylic acid (0.106 g, 0.693 mmol) and 2,3-dihydroxybenzaldehyde (0.096 g, 0.696 mmol) were dissolved in 10 mL of ethanol. A few drops of concentrated glacial acetic acid were added, and the mixture was refluxed with continuous magnetic stirring for 1 h. The resulting solution was left to evaporate at room temperature for several days, yielding a crystalline, dark red powder. The obtained product was filtered, washed with ethanol, and subsequently dissolved in a hot methanol/dichloromethane mixture. The solution was allowed to evaporate at 4 °C for a few days, resulting in stable dark red crystals of 4-{(*E*)-[(2,3-dihydroxyphenyl)­methylidene]­amino}-2-hydroxybenzoic acid (Figure S1). The synthesis pathway is illustrated in Scheme 2, the mechanism proposed for publication in Figure S2 and the characterization data are provided below. Yield: >90%; m.p.: 165.0 °C (Figure S3); ^1^H NMR (500 MHz, MeOD-*d*_4_) δ: 8.81 (s, 1H, CH=N); 7.09 (dd, 1H, *J* = 7.8 Hz, *J* = 1.4 Hz, Ar); 6.99 (dd, 1H, *J* = 7.9 Hz, *J* = 1.5 Hz, Ar); 6.90–6.89 (m, 1H, Ar); 6.85 (dd, 3H, *J* = 16.2 Hz, *J* = 8.4 Hz, Ar). ^13^C NMR: 196.37; 172.36; 163.73; 155.54; 131.52; 122.99; 122.93; 121.61; 119.51; 118.85; 106.22; 99.17. Elemental analysis calculated/found for C_14_H_11_NO_5_: C 61.51/61.53, H 4.18/4.13, N 5.17/5.16.

**Scheme 2:**
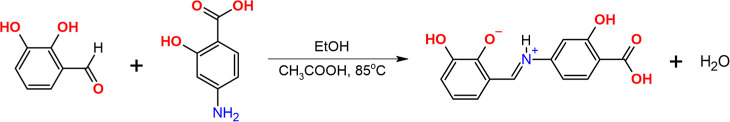
Synthesis of 4-{(*E*)-[(2,3-Dihydroxyphenyl)­methylidene]­amino}-2-hydroxybenzoic Acid

## Methods

### In Silico Prediction of Drug-Likeness, Bioavailability, and Toxicology (ADMET)

We utilized the SwissADME web tool for in silico studies on absorption, distribution, metabolism, and excretion (ADME).^67,68^ Two dimensional (2D) chemical structures of Tafamidis, Tolcapone, Diflunisal, and the Schiff bases derived from isomeric aminosalicylic acids and 2,3-dihydroxybenzaldehyde were drawn and transferred as a code of molecule, defined by canonical simplified molecular input line entry specification (SMILES). Simultaneously, the ProTox 3.0 online tool was employed to assess the expected toxicity.^69^

### Nuclear Magnetic Resonance (NMR)

The ^1^H NMR and ^13^C NMR spectra were recorded on a Bruker III Avance 500 MHz spectrometer (^1^H frequency 500.13 Hz) operated at magnetic fields of 11.7 T, using standard experimental conditions in CD_3_OD solution.

### Thermogravimetry (TG) and Differential Scanning Calorimetry (DSC)

Thermal analysis of char samples was performed using simultaneous TG-DSC analyzer Netzsch STA 449 F3 Jupiter (Germany). The sample (3 mg) was heated with a heating rate of 10 °C/min from 30 to 1000 °C in an Argon atmosphere.

### Attenuated Total Reflectance–Fourier Transform Infrared Spectroscopy (ATR–FTIR)

FTIR spectrum was measured on a PerkinElmer Spectrum 3 instrument (PerkinElmer, Waltham, USA) equipped with an attenuated total reflectance (ATR) accessory. The spectrum was recorded at room temperature in reflective mode from 4000 to 500 cm^–1^ at a resolution of 4 cm^–1^ averaging 16 scans for each measurement. The FTIR spectrum was processed and referred to their baseline using PerkinElmer Spectrum IR ver. 10.7.2 software.

### Single-Crystal X-ray Diffraction (SCXRD) Measurements and Structure Refinement

Single-crystal X-ray diffraction experiment was performed at *T* = 291(2) K (Table 1) using an Oxford Diffraction Gemini R ULTRA Ruby CCD diffractometer (λ_Cu_ = 1.54184 Å).^70^ The structure was solved and refined using the SHELX package programs.^71,72^ H-atoms from hydroxyl groups were located on a difference Fourier map and refined freely, whereas H-atoms bound to C-atoms were placed geometrically and refined using a riding model with C–H = 0.93 Å and U_iso_(H) = 1.2U_eq_(C). The hydroxyl groups (O40 and H40 atoms) have disordered orientations with refined site-occupancy factors of the disordered parts of 0.859(5) and 0.141(5). In all figures presenting the crystal structure, the disordered part of the hydroxyl group has been omitted. All interactions were found using the PLATON program.^73^ The ORTEPII^74^ and Mercury^75^ programs were used to prepare molecular graphics.

**Table 1:** Crystal Data and Structure Refinement for the Title Compound

Chemical formula	C_14_H_11_NO_5_
FW/g mol^–1^	273.24
crystal system	triclinic
space group	*P*1̅
*a*/Å	8.3459(6)
*b*/Å	10.5934(5)
*c*/Å	14.2999(6)
α/°	79.146(4)
β/°	75.887(5)
γ/°	77.109(5)
*V*/Å^3^	1183.16(12)
*Z*	4
*T*/K	291(2)
λ_Mo_/Å	0.71073
ρ_calc_/g cm^–3^	1.534
*F*(000)	568
μ/mm^–1^	0.118
θ range/°	3.33–25.00
completeness of θ/%	99.7
reflections collected	7613
reflections unique	4153 [*R*_int_ = 0.021]
data/restraints/parameters	4153/1/394
goodness of fit on *F* ^*2*^	1.049
final *R*_1_ value (*I* >2σ(*I*))	0.0507
final w*R*_2_ value (*I* >2σ(*I*))	0.1330
final *R*_1_ value (all data)	0.0626
final w*R*_2_ value (all data)	0.1421
largest diff. peak and hole/e Å^–3^	0.936 and −0.316
CCDC number	2474812

### Hirshfeld Surface, 2D Fingerprint Plot, and Energy Frameworks

Ab initio calculations of the Hirshfeld surfaces, fingerprint plots, and energy frameworks were carried out by using CrystalExplorer program (ver. 21.5).^76,77^ For both structures, the Hirshfeld surfaces were calculated with a high-resolution setting. Each molecule’s wave functions and pairwise interaction for the energy framework estimation were calculated using Tonto^78^ with the B3LYP DFT method by employing the 6-31G­(d,p) basis set, as implemented in CrystalExplorer. Interaction energy calculations between each molecule and its chemical neighborhood were performed, generating a cluster within a radius of 3.8 Å. Energy frameworks, shown as colored cylinders between interacting molecular centroids, illustrate selected directional energies. A tube size of 50 and a 10 kJ/mol energy cutoff were used for clarity.

### Theoretical Calculations

Quantum chemical calculations of the electrostatic potential (MEP) and Frontier molecular orbitals (FMO) were performed using the Gaussian 09W software.^79^ All calculations were conducted at the DFT/B3LYP level of theory with the 6-31++G­(d,p) basis set. The molecular geometry of the two independent molecules in the asymmetric unit of the obtained compound was optimized in the gas phase. The DFT-derived key bond lengths for 4-{(*E*)-[(2,3-dihydroxyphenyl)­methylidene]­amino}-2-hydroxybenzoic acid were compared with available crystallographic data.^80,81^ The computational and experimental bond lengths were determined as follows: N10–C11 (1.337 Å/DFT and 1.312 Å/XRD), N10–C4 (1.403 Å/DFT and 1.412 Å/XRD), C2–O20 (1.345 Å/DFT and 1.349 Å/XRD), C7–O8 (1.245 Å/DFT and 1.231 Å/XRD), C7–O9 (1.325 Å/DFT and 1.311 Å/XRD), C13–O18 (1.274 Å/DFT and 1.284 Å/XRD), C14–O19 (1.357 Å/DFT and 1.370 Å/XRD), N30–C31 (1.411 Å/DFT and 1.413 Å/XRD), N30–C24 (1.330 Å/DFT and 1.314 Å/XRD), C22–O40 (1.343 Å/DFT and 1.345 Å/XRD), C27–O28 (1.348 Å/DFT and 1.309 Å/XRD), C27–O29 (1.234 Å/DFT and 1.230 Å/XRD), C33–O38 (1.287 Å/DFT and 1.284 Å/XRD), C34–O39 (1.359 Å/DFT and 1.365 Å/XRD). The results demonstrate that the computational and experimental data are in agreement. The molecular electrostatic potential, highest occupied molecular orbital (HOMO), and lowest unoccupied molecular orbital (LUMO) were visualized using GaussView (ver. 5).^82^

### Molecular Docking Studies

All molecular fingerprint calculations were performed using Python 3.9.23 and the RDKit cheminformatics library (via the conda-forge distribution).^83^ Compounds were represented as SMILES strings and converted to molecular objects by using Chem.MolFromSmiles. Two types of fingerprints were generated: RDK fingerprints using Chem.RDKFingerprint with parameters minPath = 1, maxPath = 5, and fpSize = 1024, and MACCS structural keys, represented as 166-bit binary vectors generated via MACCSkeys.GenMACCSKeys. Structural similarity between molecule pairs was quantified using the Tanimoto similarity coefficient computed with DataStructs.TanimotoSimilarity.

All docking simulations were performed using AutoDock 4.2.1 and Vina 1.1.2 program.^84^ From the Protein Data Bank (PDB; http://www.wwpdb.org/), we selected the crystal structure complex of TTR with bound tolcapone (PDB no. 4D7B with 1.15 Å resolution)^85^ to constitute the receptor model for human TTR. The CIF file of obtained compound was utilized in the study. The receptor structure was prepared according to the protocols provided by Autodock. Briefly, the protein and ligand were separated, water molecules were removed, polar hydrogen atoms were added, and Gasteiger charges were assigned. The resulting protein structure was saved in PDBQT format by using AutoDock Tools. Seven rotatable bonds were selected by default. The gridbox was selected to occupy the key amino acid residues in the active site with the following parameters: center_*x* = 0.00, center_*y* = 0.00, center_*z* = −4.30, size_*x* = 26, size_*y* = 26, and size_*z* = 26. The energy range and exhaustiveness were set to 8 and 16, respectively. PyMOL 3.1.3 was used to choose the best binding poses and export them to the PDB file.^86^ Visualization of docking results on the enzyme surface and 2D interaction diagrams from the PDB file were generated using BIOVIA Discovery Studio Visualizer V24.^87^

## Results and Discussion

### ADMET Results

Given that Schiff bases are considered promising candidates as enzyme inhibitors, we first conducted an in silico analysis of their pharmacokinetic properties using SwissADME to assess their overall suitability as potential drug candidates. This tool offers a straightforward yet valuable method for characterizing such compounds and is increasingly used for the preliminary evaluation of pharmacokinetic profiles.^88,89^ The predicted properties of the synthesized compounds are summarized in Table S1. Examination of drug-likeness parameters using the bioavailability radar (Figure 1a–d) shows that the Schiff bases derived from isomeric aminosalicylic acids and 2,3-dihydroxybenzaldehyde, along with three reference drugs (Tafamidis, Diflunisal, and Tolcapone), exhibit favorable lipophilicity and water solubility—key properties for effective absorption and subsequent metabolism.^90–92^

**Figure 1:**
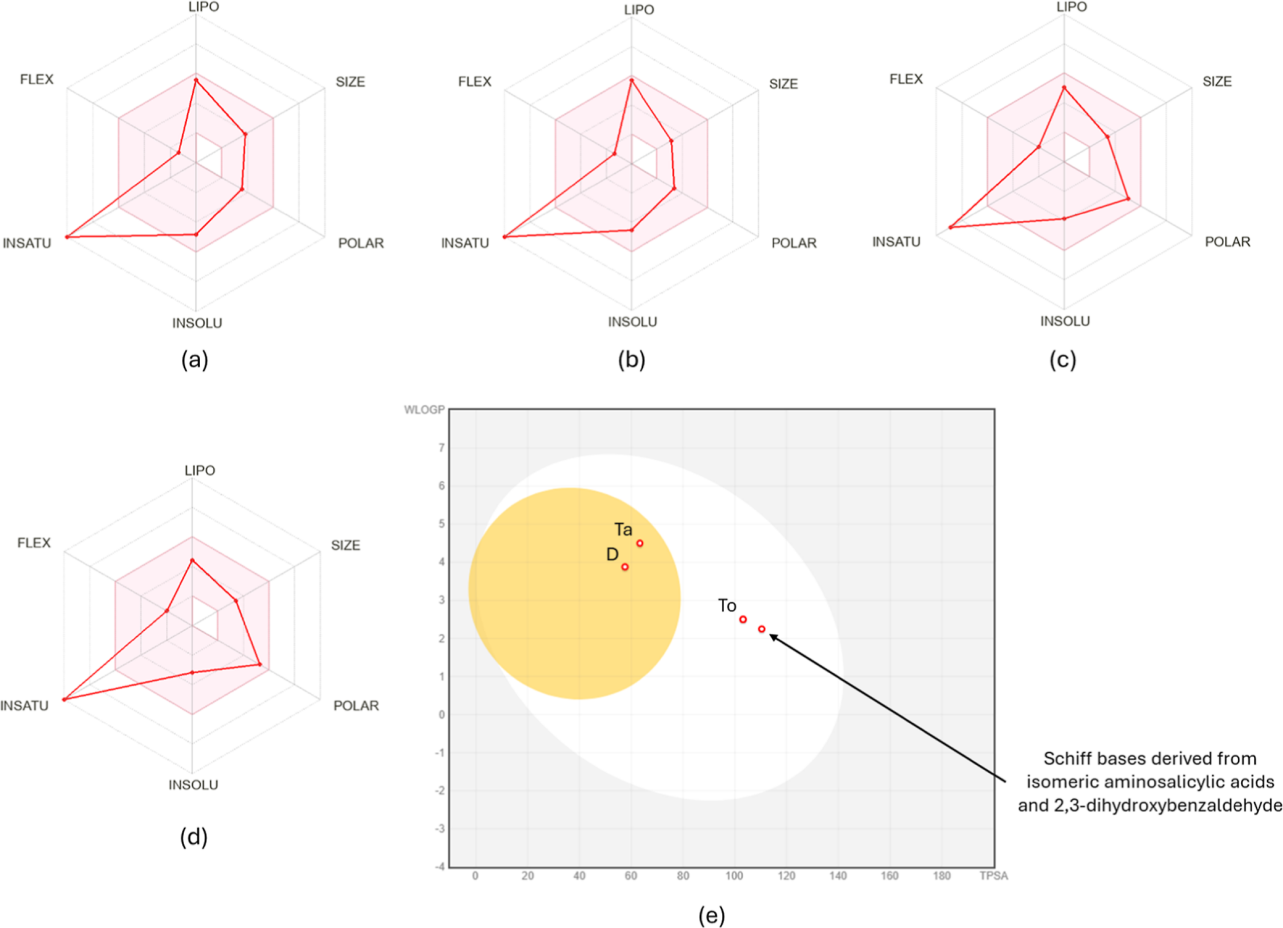
Bioavailability radars for (a) Tafamidis (Ta), (b) Tolcapone (To), (c) Diflunisal (D), (d) Schiff bases derived from aminosalicylic acids and 2,3-dihydroxybenzaldehyde, and (e) BOILED-Egg plot.

Analysis of the BOILED-Egg plot (Figure 1d) reveals that the Schiff bases fall within the white ellipse, suggesting less favorable pharmacokinetic properties compared to Tafamidis and Diflunisal, but comparable to Tolcapone, an established TTR stabilizer. Although the Schiff bases are unable to cross the blood–brain barrier, they still demonstrate promising potential as TTR inhibitors with high gastrointestinal absorption.

The results of toxicity predictions using the ProTox 3.0 online tool are presented in Table S2. The Schiff bases fall under Class IV of acute oral toxicity, indicating that according to US EPA classification, its LD_50_ value is 1500 mg/kg. Interestingly, the toxicity prediction results suggest that the obtained compound may act as a transthyretin inhibitor.

### Thermal Characterization

Thermal analysis (DSC/TG) of the obtained compound revealed a single endothermic peak associated with melting and initial decomposition, observed in the temperature range of 142–205 °C, with a maximum at 170 °C (Figure 2). This thermal event was accompanied by a 22% mass loss on the TG curve, confirming that decomposition occurred simultaneously with melting. The melting point determined by using a Buchi apparatus was 165 °C, consistent with the DSC peak observed at 168 °C. In addition to the initial decomposition stage, the TG/DTG data indicated two further thermal degradation steps. The second decomposition stage occurred between 205 and 255 °C, with a DTG maximum at 215 °C, and the third stage was observed between 255 and 318 °C, reaching a maximum at 285 °C. Each of these two later steps was associated with an additional 7% mass loss, indicating a gradual and multistep decomposition process. The DSC trace showed a broad, weak endothermic signal in the higher temperature range, corresponding to these two later decomposition stages, with a maximum effect at approximately 280 °C. These results suggest that the thermal decomposition of the compound proceeds in at least three distinct steps, with the first step overlapping with melting and the subsequent steps representing progressive breakdown of the compound’s structure.

**Figure 2:**
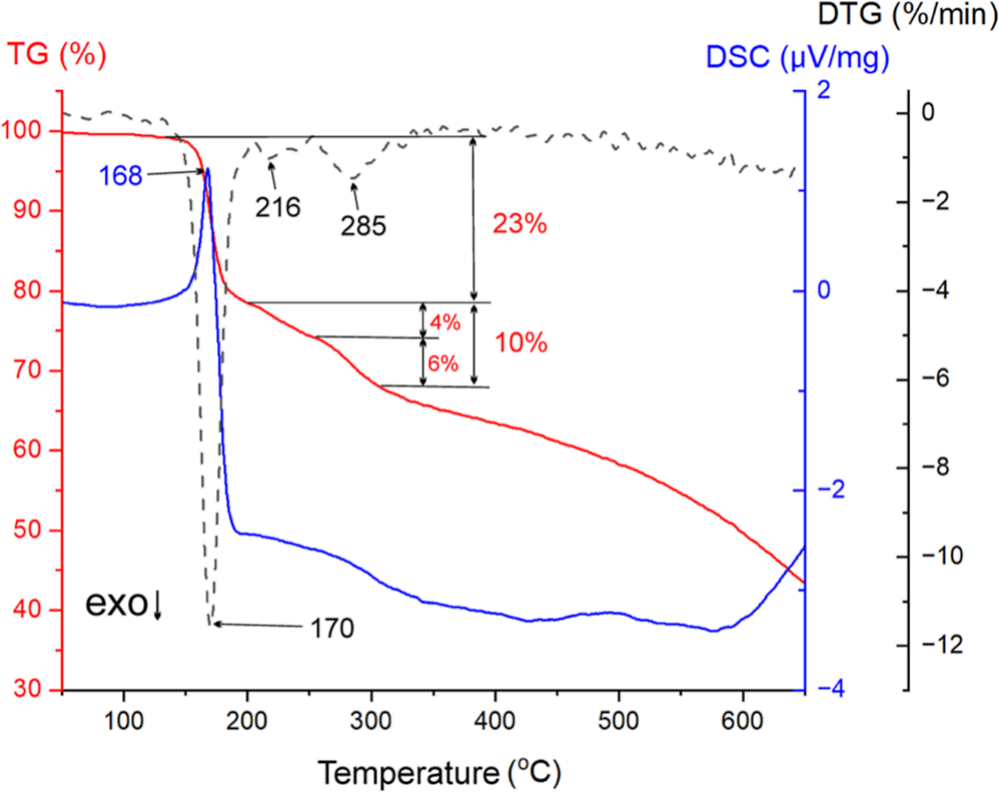
TG curve (dashed) and DSC curve (solid) of the 4-{(*E*)-[(2,3-dihydroxyphenyl)-methylidene]­amino}-2-hydroxybenzoic acid.

### Spectral Characterization

The ATR-FTIR spectrum of the title compound is presented in Figure 3. The absence of characteristic bands for primary amines (N–H) in 4-aminosalicylic acid^93^ and the carbonyl C=O stretching band typical of 2,3-dihydroxybenzaldehyde,^94^ along with the presence of a single overlapped band at 1622 cm^–1^, which corresponds to azomethine (−HC=N−) stretching vibrations,^95–97^ suggests the formation of the Schiff base. A band corresponding to carboxylic C=O stretching vibrations is observed at 1651 cm^–1^, which is lower than the typical range for free carbonyl groups (1760–1690 cm^–1^). This shift can be attributed to the involvement of carboxyl groups in strong hydrogen-bonding interactions.^98,99^ Additionally, weak broad bands at 3400 cm^–1^ and 3265 cm^–1^ correspond to the O–H stretching and N–H stretching vibrations, respectively. Both precursor compounds, 4-aminosalicylic acid and 2,3-dihydroxybenzaldehyde, exhibit strong, broad O–H stretching bands. However, the disappearance of these bands in the Schiff base suggests that hydroxyl groups are involved in extensive hydrogen bonding, likely forming robust molecular synthons.^93,94^ A strong band observed at 1208 cm^–1^ is likely attributed to C–O stretching vibrations.^100,101^ Additionally, the band at 3062 cm^–1^ is attributed to C–H stretching vibrations, while the bands at 850 and 734 cm^–1^ correspond to C–H out-of-plane bending vibrations. Several bands in the 1611–1430 cm^–1^ range are associated with aromatic C = C stretching vibrations, confirming the aromatic system.

**Figure 3:**
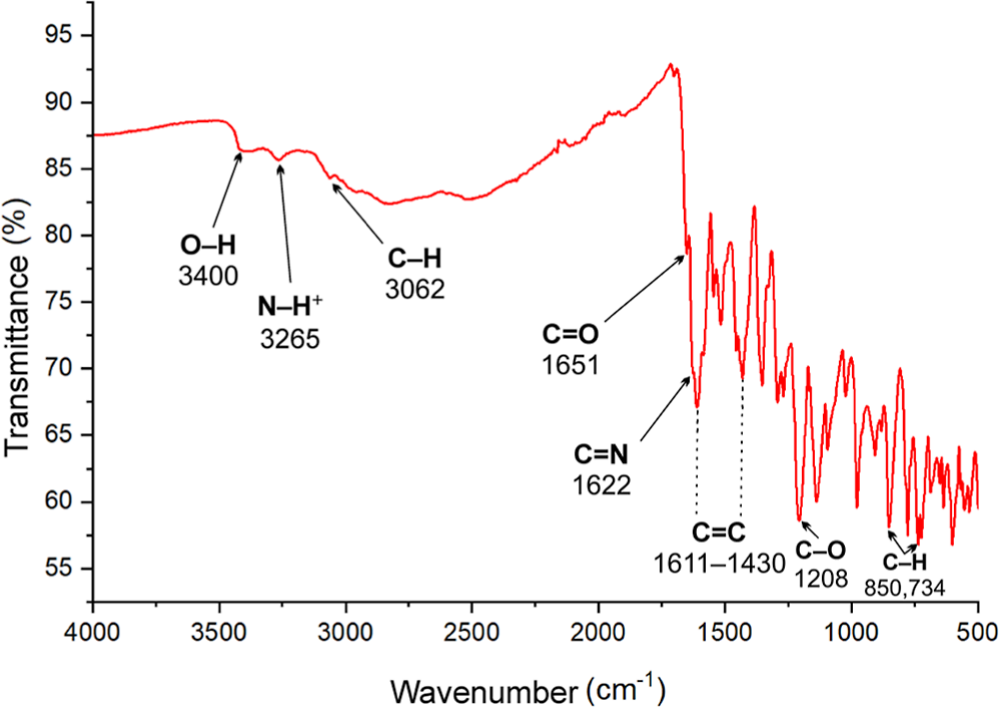
ATR-FTIR spectrum of the 4-{(*E*)-[(2,3-dihydroxyphenyl)-methylidene]­amino}-2-hydroxybenzoic acid.

### Crystal Structure and Intermolecular Interactions

Single-crystal X-ray diffraction measurements show that the obtained compound crystallizes in the *P̅*1 triclinic space group, with two molecules of 4-{(*E*)-[(2,3-dihydroxyphenyl)­methylidene]­amino}-2-hydroxybenzoic acid in the asymmetric unit, as depicted in Figure 4. Crystal data and structure refinement are given in Table 1.

**Figure 4:**
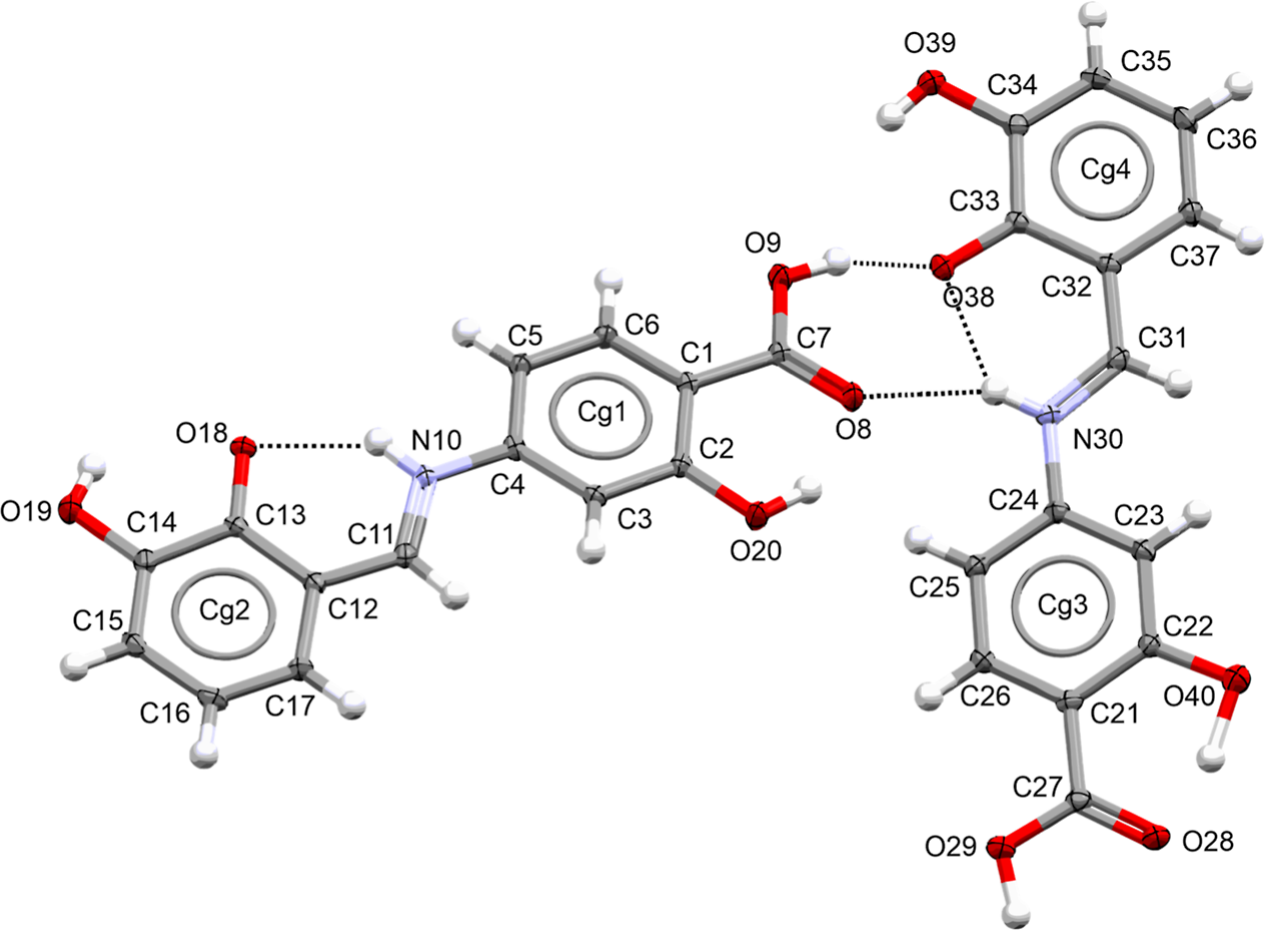
Asymmetric unit of the title compound with atom labels. Displacement ellipsoids at 50% probability (hydrogen bonds shown as dashed lines; Cg represents the center of gravity of the aromatic rings).

In the crystal structure of the compound, an intramolecular proton transfer O^–^···H–N^+^ between the hydroxyl group and the azomethine nitrogen atom is observed, leading both conformers to adopt the keto form in the zwitterionic imine state. To further validate this observation, we conducted a comparative analysis of geometric parameters, including bond lengths and valence angles, using structures of related phenol–imine compounds from the CSD.^102^ The C13–O18 bond length within the hydroxyl group is shorter [1.284 Å] compared to the enol range of 1.341–1.359 Å. Similarly, the C11–C12 bond length is reduced [1.401 Å] in comparison to the enol range of 1.434–1.454 Å. The C11–N10 bond length [1.312 Å] falls within the typical keto form range of 1.288–1.317 Å. The observed shortening of the C13–O18 and C11–C12 bonds, along with the elongation of the C11–N10 bond, suggests proton transfer, resulting in the formation of an intramolecular N10^+^–H10···O18^–^ hydrogen bond. This interaction confers a keto character to the C13–O18 bond and a single-bond character to the C11–C12 bond.

The crystal structure also reveals that two conformers which interact with each other through the N_(imine)_–H···O_(carboxyl)_ [*d*(H30···O8) = 2.62 Å and < (N30–H30···O8) = 134.5°, and *d*(H10···O28) = 2.68 Å and < (N10–H10···O28) = 131.4°] and O_(carboxyl)_–H···O_(hydroxyl)_ [*d*(H9···O38) = 1.72 Å and < (O9–H9···O38) = 159.6°, and *d*(H29···O18) = 1.67 Å and < (O29–H29···O18) = 169.2°] hydrogen bonds, forming a cyclic heterosynthon (1). Hydrogen-bond details are listed in Table S3. The Hirshfeld surface for the aforementioned synthon, showing several red and pale red areas that indicate close contacts between atoms on the surface, is presented in Figure 5.

**Figure 5:**
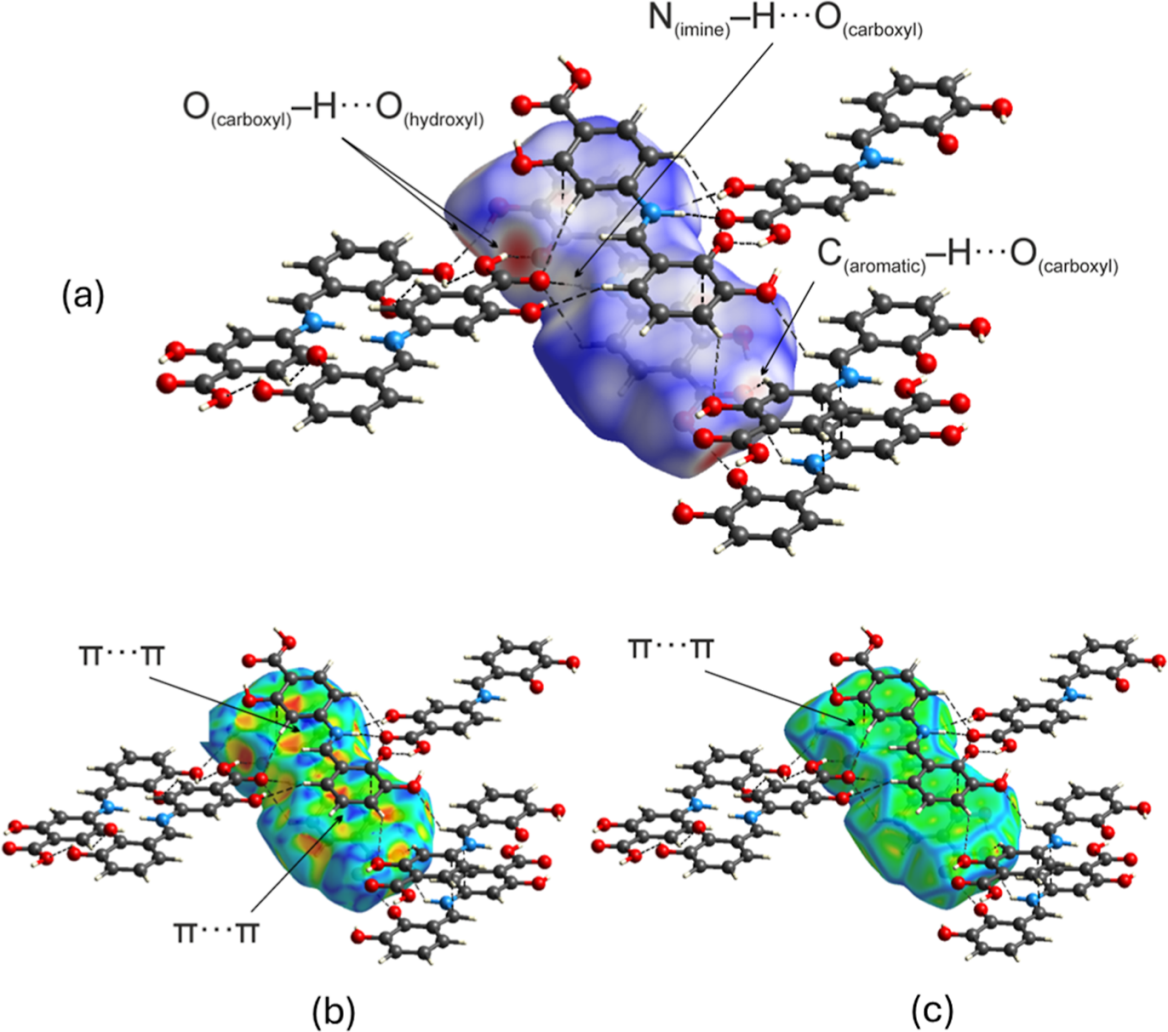
Hirshfeld surfaces of the molecules in the asymmetric unit of the unit cell generated using (a) *d*_norm_, (b) shape index, and (c) curvedness parameters.

Creating a cyclic heterosynthon results in forming a zigzag chain motif along the crystallographic [0 0 1] direction as presented in Figure 6a. Adjacent zigzag chains interact with each other through another heterosynthon (2) consisting of O_(hydroxyl)_–H···O_(carboxyl)_ [d­(H19···O9) = 2.43 Å and < (O19–H19···O9) = 133.7°] and C_(aromatic)_–H···O_(hydroxyl)_ [*d*(H6···O18) = 2.60 Å and < (C6–H6···O18) = 139.5°] hydrogen bonds. Additionally, extra stabilizing O_(hydroxyl)_–H···O_(carboxyl)_ and C_(aromatic)_–H···O_(carboxyl)_ [*d*(H25···O8) = 2.67 Å and < (C25–H25···O8) = 121.4°] hydrogen bonds are also observed [*d*(H39···O19) = 2.14 Å and < (C6–H6···O18) = 145.9°], as shown in Figure 6b, forming a three-dimensional layer-type network. This network is illustrated in Figure 6c. The π–π stacking interactions, with Cg···Cg distances ranging from 3.709 to 3.719 Å between conformer molecules in adjacent zigzag chains, together with a C–O···π interaction, provide additional stabilization to the three-dimensional, layer-type network. These interactions are detailed in Tables S4 and S5.

**Figure 6:**
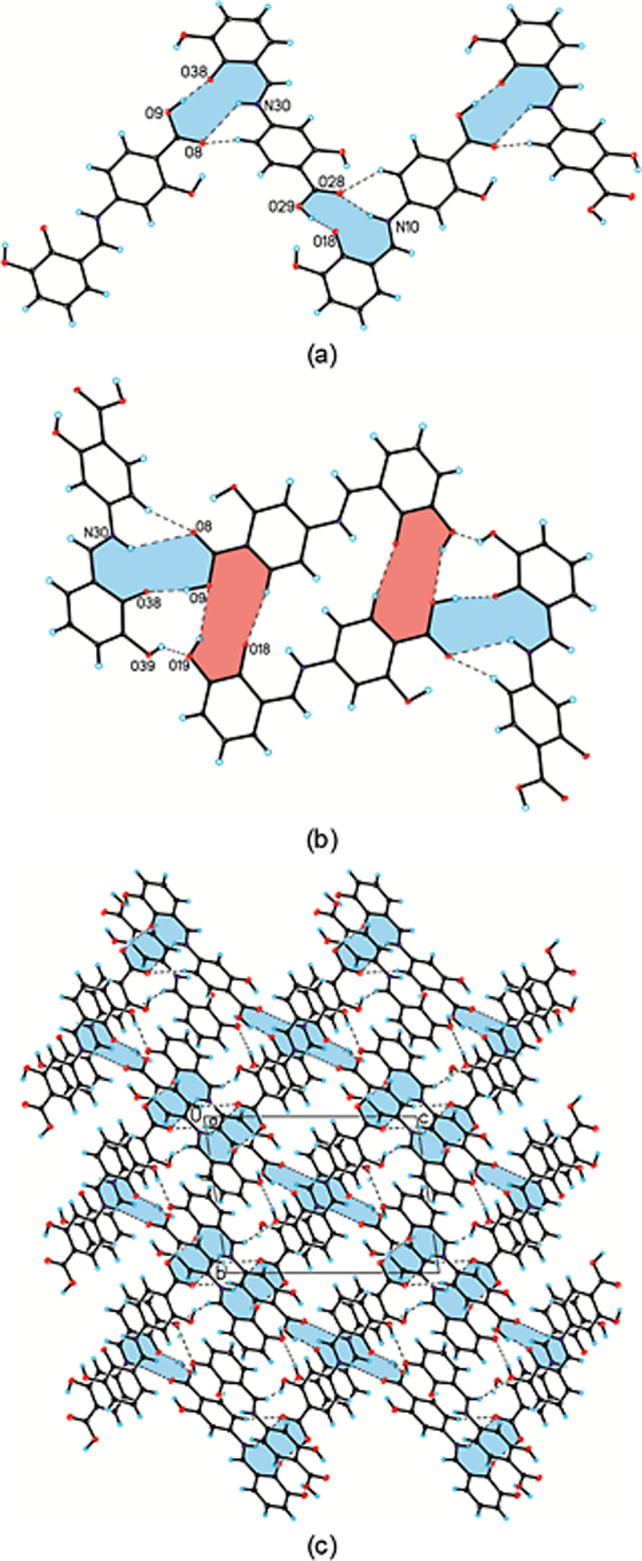
View of (a) the chain motif linked via heterosynthon (1), (b) two adjacent chains linked via heterosynthon (2), and (c) crystal packing along the *c*-axis. Hydrogen bonds are shown as black dashed lines; heterosynthons (1) and (2) are highlighted in blue and orange, respectively.

The Hirshfeld surface mapped with the shape index function shows the presence of ‘bow-tie’ patterns, confirming aromatic π–π stacking interactions, as presented in Figure 7a. Additionally, the large green flat region between molecules provides further evidence of aromatic stacking interactions, as shown in Figure 7b. These findings are consistent with results reported by Gumus et al.^103^ and McKinnon et al.^104^

**Figure 7:**
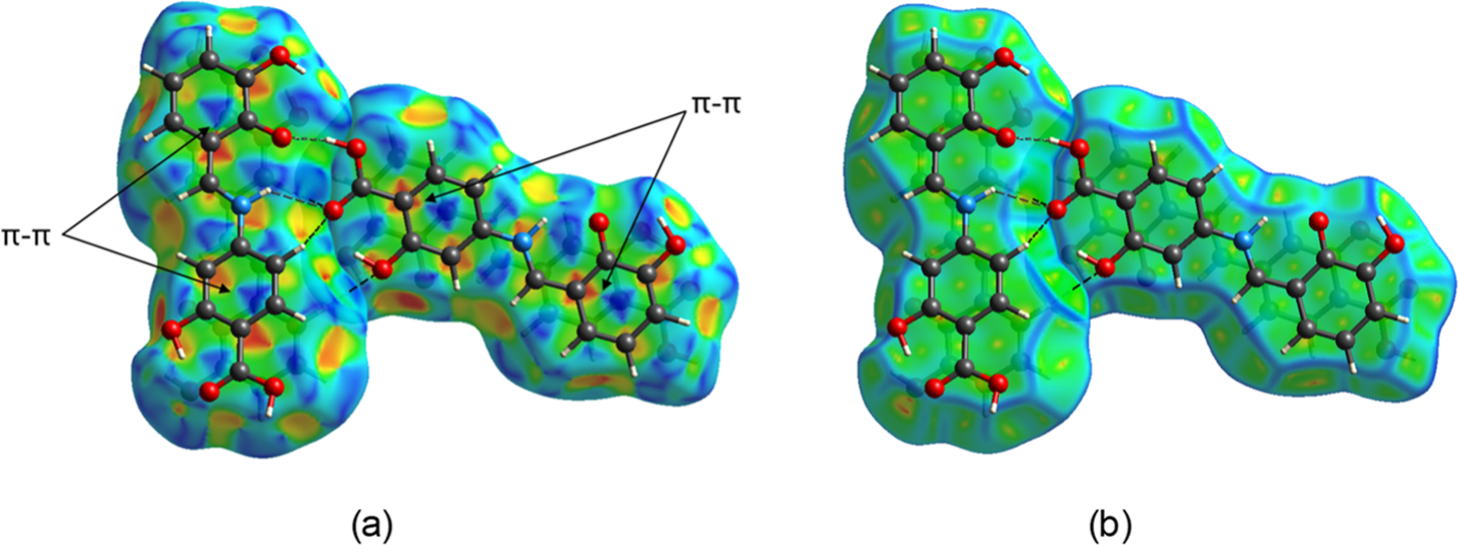
Hirshfeld surface of two conformers mapped over (a) shape index and (b) curvedness, with hydrogen bonds shown as black dashed lines.

Analysis of decomposed 2D fingerprint plots (Figure 8) reveals the key intermolecular interactions within the crystal structure of 4-{(*E*)-[(2,3-dihydroxyphenyl)­methylidene]­amino}-2-hydroxybenzoic acid. The most significant are the O···H/H···O contacts, accounting for 34.2% of the total Hirshfeld surface. These appear as sharp spikes in the plots and correspond to heterosynthons (1 and 2), formed by C–H···O and N–H···O hydrogen bonds, visible as red spots on the *d*_norm_-mapped Hirshfeld surface. van der Waals interactions (H···H contacts) represent the second-largest contribution, at 33.2%. Additionally, C···C and C···H/H···C contacts, associated with π–π stacking, contribute 14.0% and 10.3%, respectively.

**Figure 8:**
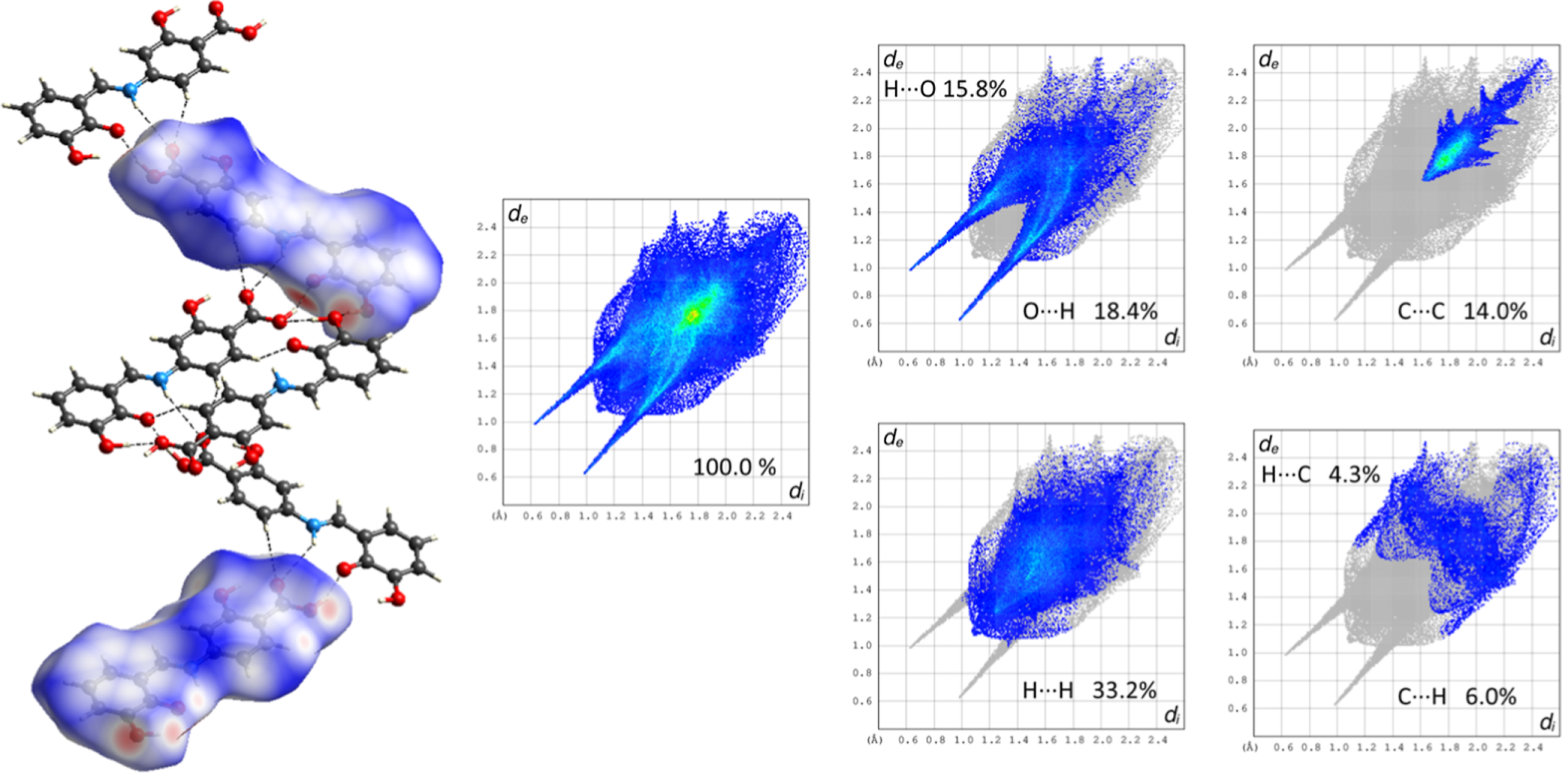
Fingerprint plots illustrating the percentage contribution of the most prominent interactions in the crystal lattice of the obtained compound.

The total interaction energies indicate that heterosynthon (1) (−53.0 and −52.7 kJ/mol) is significantly stronger than heterosynthon (2) (−36.3 kJ/mol), confirming that the zigzag chain is the dominant motif in the crystal lattice, as presented in Figure 9a and 9b. The interactions between Schiff base molecules involved in stacking interactions are −49.4 kJ/mol for stacking within conformer 1 molecules and −50.2 kJ/mol for stacking between conformer 2 molecules, suggesting the key role of dispersion forces in the arrangement of molecules in the crystal lattice, as shown in Figure 9c and 9d. Selected interaction energies are summarized in Figure S4. Energy frameworks showing electrostatic, dispersion, and total energies for a molecular cluster are presented in Figure S5.

**Figure 9:**
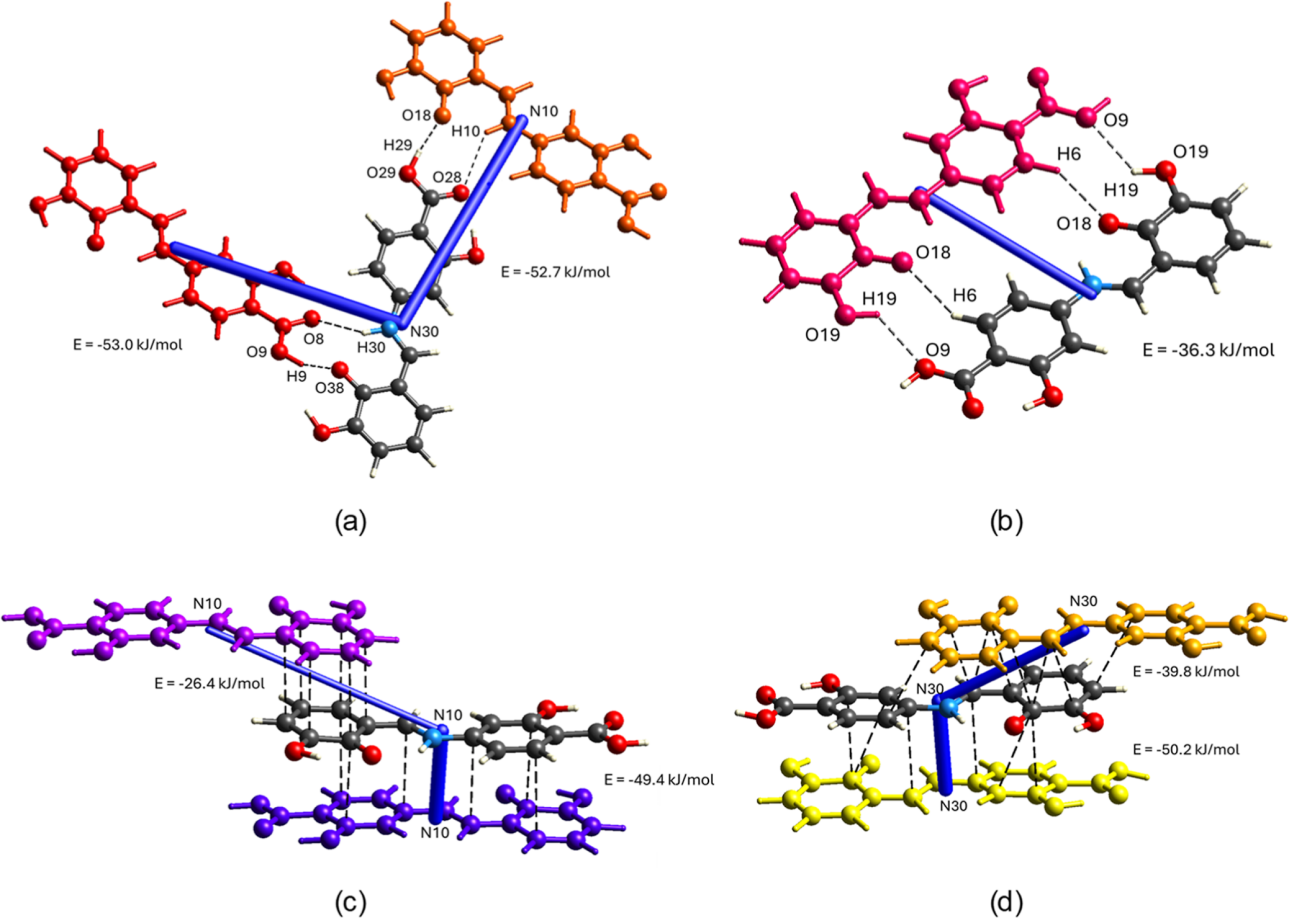
Energy representation of molecular interactions (a–d) in the crystal of the title compound, showing the central (noncolored) and surrounding (colored) molecules.

### Theoretical Studies

Structural analysis of the obtained compound, which highlighted a significant contribution of molecular synthons and π–π stacking interactions, combined with the general understanding of the broad range of Schiff bases used in optoelectronic applications,^105–108^ led us to suspect that this crystalline structure may possess similar potential. To investigate its electro-optic properties, we performed DFT calculations using the B3LYP/6-31++G­(d,p) level to obtain the molecular electrostatic potential (MEP), frontier molecular orbitals (HOMO, LUMO), and energy gap (Δ*E*).

First, we generated the MEP, which provides a visual representation of the relative polarity of the molecule.^109^ Different colors correspond to varying electrostatic potential values. Red and yellow areas, characterized by a high electron density (negative charge), indicate potential electrophilic attack sites. In contrast, blue regions, with a lower electron density, suggest potential nucleophilic attack sites. Green areas represent the electrostatically neutral parts of the molecule. The presence of yellow regions near hydroxyl groups indicates their higher electronegativity, suggesting that they may act as acceptor centers. Blue regions observed near the azomethine group and the hydrogen atom of the carboxyl group are more electropositive, potentially serving as donor centers. Consequently, the MEP analysis reveals that the molecules of the obtained compound exhibit donor–acceptor character. Considering that the distribution of the electrostatic potential can influence charge transport, the obtained compound can demonstrate key properties for optoelectronic applications. The molecular electrostatic potential surface for the asymmetric unit of 4-{(*E*)-[(2,3-dihydroxyphenyl)­methylidene]­amino}-2-hydroxybenzoic acid crystal is presented in Figure 10.

**Figure 10:**
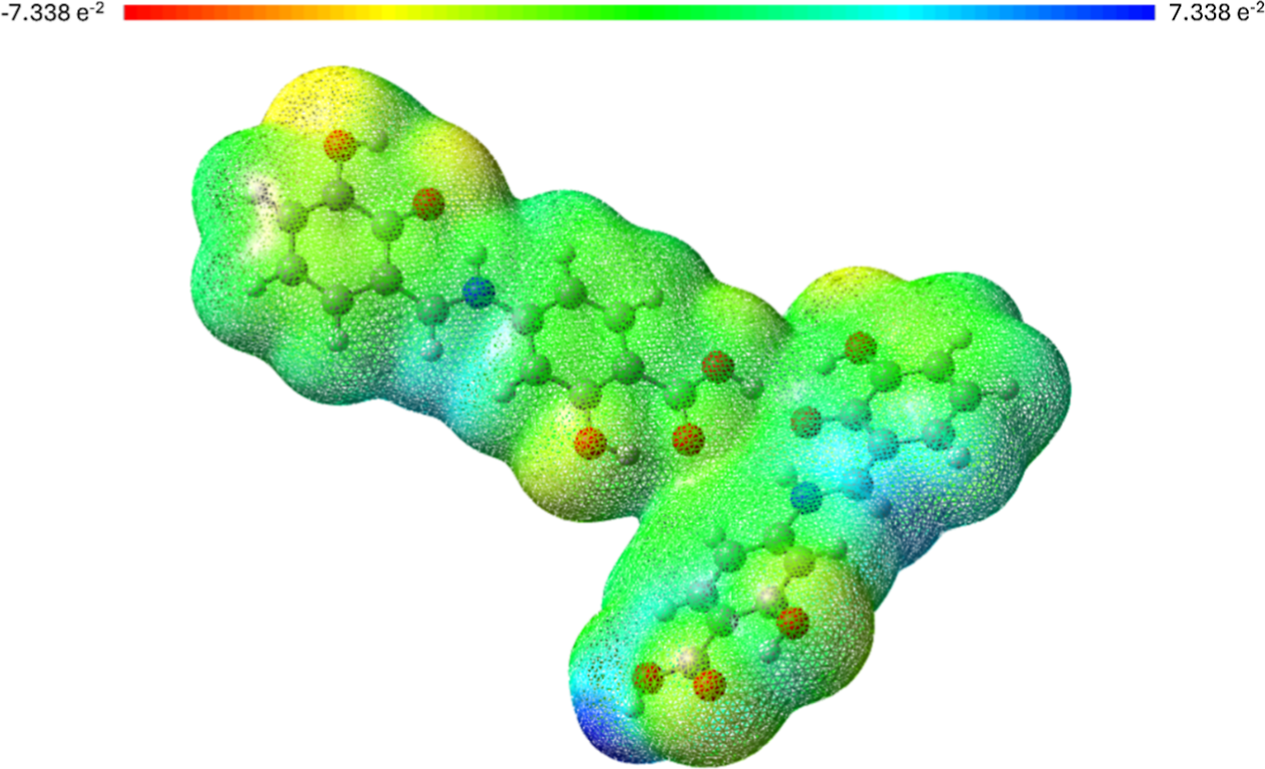
Molecular electrostatic potential surface of the asymmetric unit of the title compound. Color scale ranges from −7.338 a.u (red) to 7.338 a.u (blue).

The frontier molecular orbitals define the charge transfer pathways within the system and play critical roles in determining its electrical and optical properties. The highest occupied molecular orbital (HOMO) is typically considered nucleophilic and capable of donating electrons, while the lowest unoccupied molecular orbital (LUMO) serves as an electrophilic site, accepting electrons. The energy gap between the HOMO and LUMO governs the overall charge transfer interaction within the molecule and is key in determining its electrical transport properties.^110–112^ A large HOMO–LUMO energy gap indicates low chemical reactivity and high kinetic stability of the molecular components, based on their electronic transition sites.^108^ In the obtained compound, the HOMO and LUMO are localized on two distinct molecules within the asymmetric unit, suggesting charge transfer along the N_(imine)_–H···O_(carboxyl)_ and O_(carboxyl)_–H···O_(hydroxyl)_ hydrogen-bond heterosynthon. This transfer occurs from the HOMO of the donor molecule to the unoccupied LUMO of the acceptor molecule, as illustrated in Figure 11. The calculated energy gap (Δ*E*) between the HOMO and LUMO is 2.341 eV. The relatively low Δ*E* value indicates that the obtained compound exhibits low chemical reactivity and high kinetic stability.

**Figure 11:**
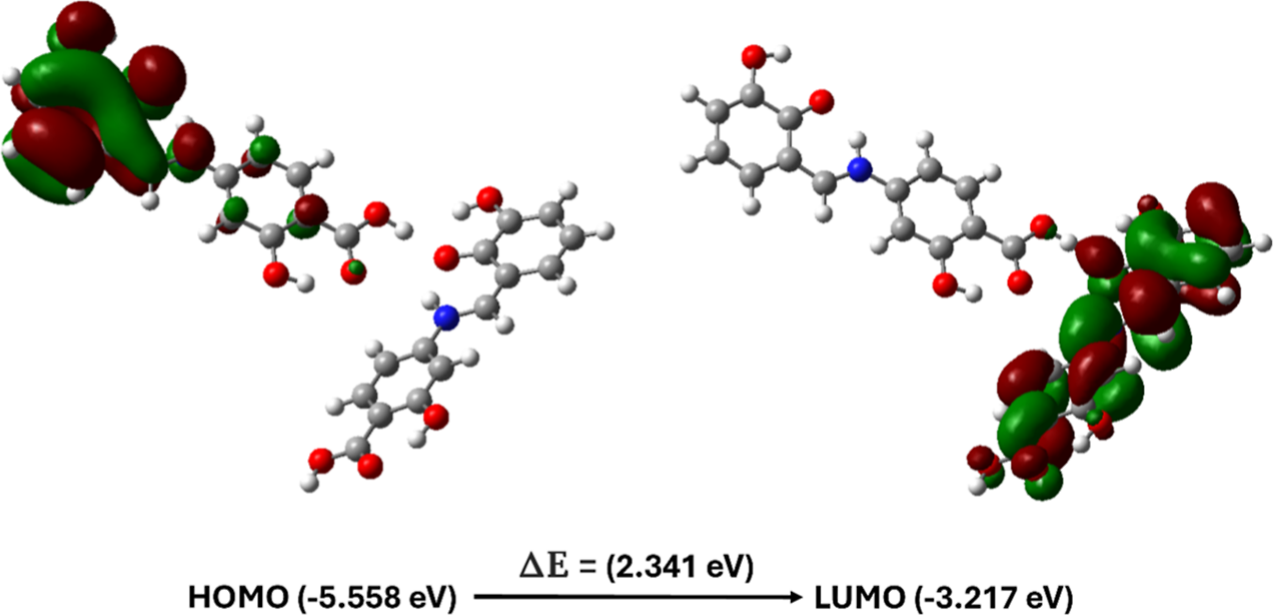
Spatial HOMO–LUMO plots and associated energy gap (Δ*E*) for the asymmetric unit of the 4-{(*E*)-[(2,3-dihydroxyphenyl)­methylidene]­amino}-2-hydroxybenzoic acid crystal.

Global chemical reactivity, derived from the HOMO and LUMO energy values, provides key descriptors such as hardness (η), electrophilicity index (ω), softness (s), chemical potential (μ), electronegativity (χ), and ionization potential (I) (Table 2). These descriptors play an important role in quantitative structure–activity relationship (QSAR) modeling, serving as fundamental factors in drug design and helping to predict the biological activity and efficacy of potential drug candidates.^113,114^

**Table 2:** Global Chemical Reactivity Descriptors for the Asymmetric Unit of the 4-{(*E*)-[(2,3-Dihydroxyphenyl)­methylidene]­amino}-2-hydroxybenzoic Acid Crystal

descriptor	value	unit
chemical hardness (η)	1.170	eV
chemical softness (*s*)	0.427	eV^–1^
chemical potential (μ)	–4.387	eV
electronegativity (χ)	4.387	eV
electrophilicity index (ω)	8.222	eV
ionization potential (*I*)	5.557	eV
electron affinity (*A*)	3.216	eV
dipole moment (μ)	6.799	debye (D)

The dipole moment for the molecules in the asymmetric unit is 6.799 D, indicating significant molecular polarity. This suggests the potential to form strong intermolecular interactions, particularly with highly polar species. Such interactions may play a crucial role in the compound’s binding with biomolecules, influencing its potential applications in drug design.

### Molecular Docking Studies

The dissociation of TTR into monomeric species is a crucial step in the formation of amyloid fibrils, which are linked to transthyretin amyloidosis.^115^ Therefore, identifying small molecules—such as thyroxine and salicylic acid derivatives or diflunisal analogues—that can stabilize the tetrameric structure of TTR is of great therapeutic importance.^116–118^

First, to assess whether 4-{(*E*)-[(2,3-dihydroxyphenyl)­methylidene]­amino}-2-hydroxybenzoic acid is a good candidate as a TTR inhibitor, we conducted a screening analysis of its structural similarity to known TTR stabilizers. To this end, we employed MACCS keys, which are predefined, substructure-based binary fingerprints that primarily reflect the presence or absence of specific functional groups, and RDK fingerprints, which are path-based and encode topological atom sequences, thereby capturing the overall molecular shape and atom connectivity. Structural similarity was quantified using the Tanimoto coefficient, a widely used metric for comparing molecular fingerprints.^119^ These results are presented in Table 3.

**Table 3:** Tanimoto Coefficient-Based Structural Similarity between the Obtained Compound and Known TTR Stabilizers

structural similarity (Tanimoto coefficient)							
Diflunisal		Tolcapone		Tafamidis		salicylic acid	
MACCS	RDK	MACCS	RDK	MACCS	RDK	MACCS	RDK
0.57	0.42	0.62	0.35	0.30	0.22	0.59	0.54

The compound exhibited comparable structural similarity to both diflunisal (MACCS: 0.57; RDK: 0.42) and tolcapone (MACCS: 0.62; RDK: 0.35), both of which are approved TTR-stabilizing drugs. These results suggest partial structural resemblance to both reference compounds with slightly greater similarity in functional group composition to tolcapone. A similar approach, based on structural similarity and the Tanimoto coefficient, was previously employed by Yokoyama et al.^119^ to identify potential TTR-binding compounds, further supporting the relevance of this strategy in early-stage screening.

Given the structural similarity to tolcapone, the TTR protein structure cocrystallized with tolcapone was selected as the basis for subsequent docking studies. As a validation step, tolcapone was redocked into the experimental binding site, and the resulting poses were compared to its crystallographic conformation. Notably, the top two docking poses exhibited a reversed binding orientation relative to that of the experimental pose. Therefore, the third-ranked conformation, showing the highest conformational similarity and best alignment of key interactions with the crystal structure, was selected as the reference for further comparison. The visualization of the selected conformations of the experimental, redocked, and obtained compound is presented in Figure 12.

**Figure 12:**
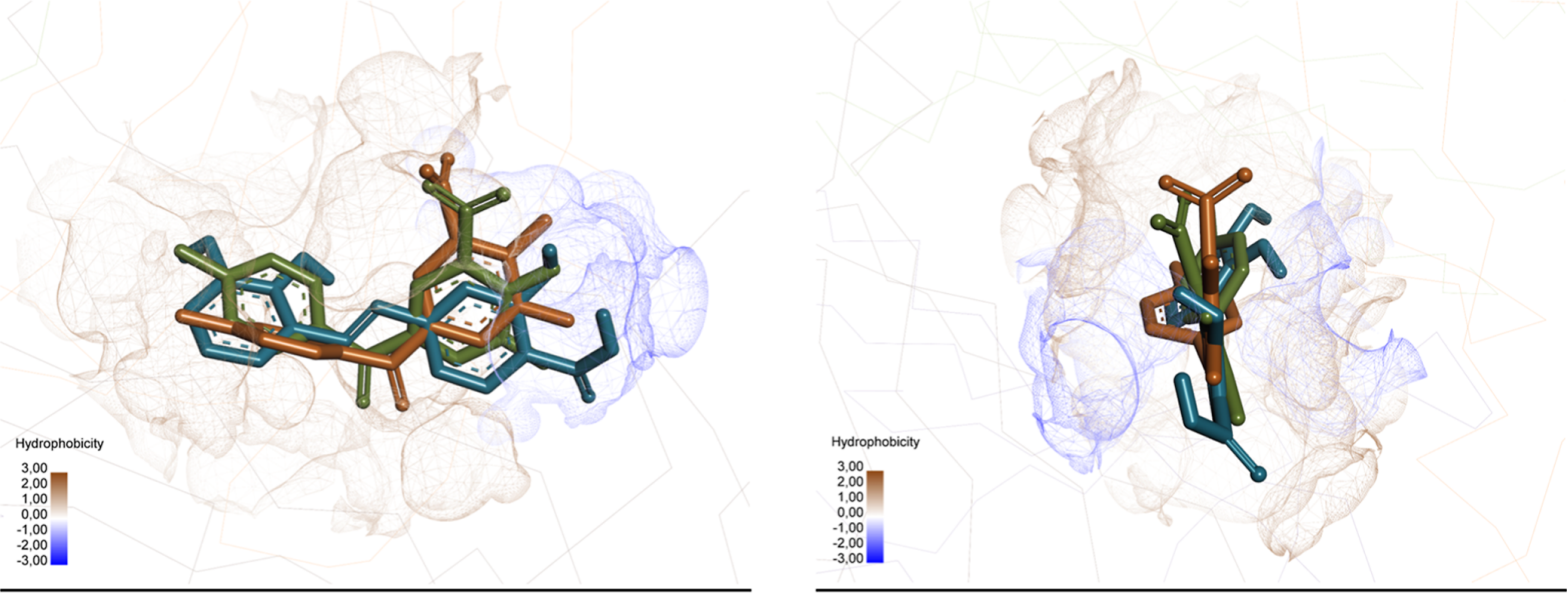
Visualization of the selected conformations of the experimental (olive) and redocked (brown) of Tolcapone and obtained compound (cyan) in the active site of TTR.

Subsequently, the designed compound was docked into the same TTR structure (Figure 13), and its predicted binding mode was evaluated with respect to both the experimental and the validated docking poses of tolcapone. The top-ranked pose of the compound (pose 1) was found to exhibit sufficient similarity in orientation to both reference conformations and was therefore selected for further analysis. At this stage, a comparative assessment of molecular interactions was carried out across all three binding modes: the experimental tolcapone pose, its redocked conformation, and the docked pose of the designed compound to identify similarities and differences in interaction patterns within the TTR active site (Table 4).

**Figure 13:**
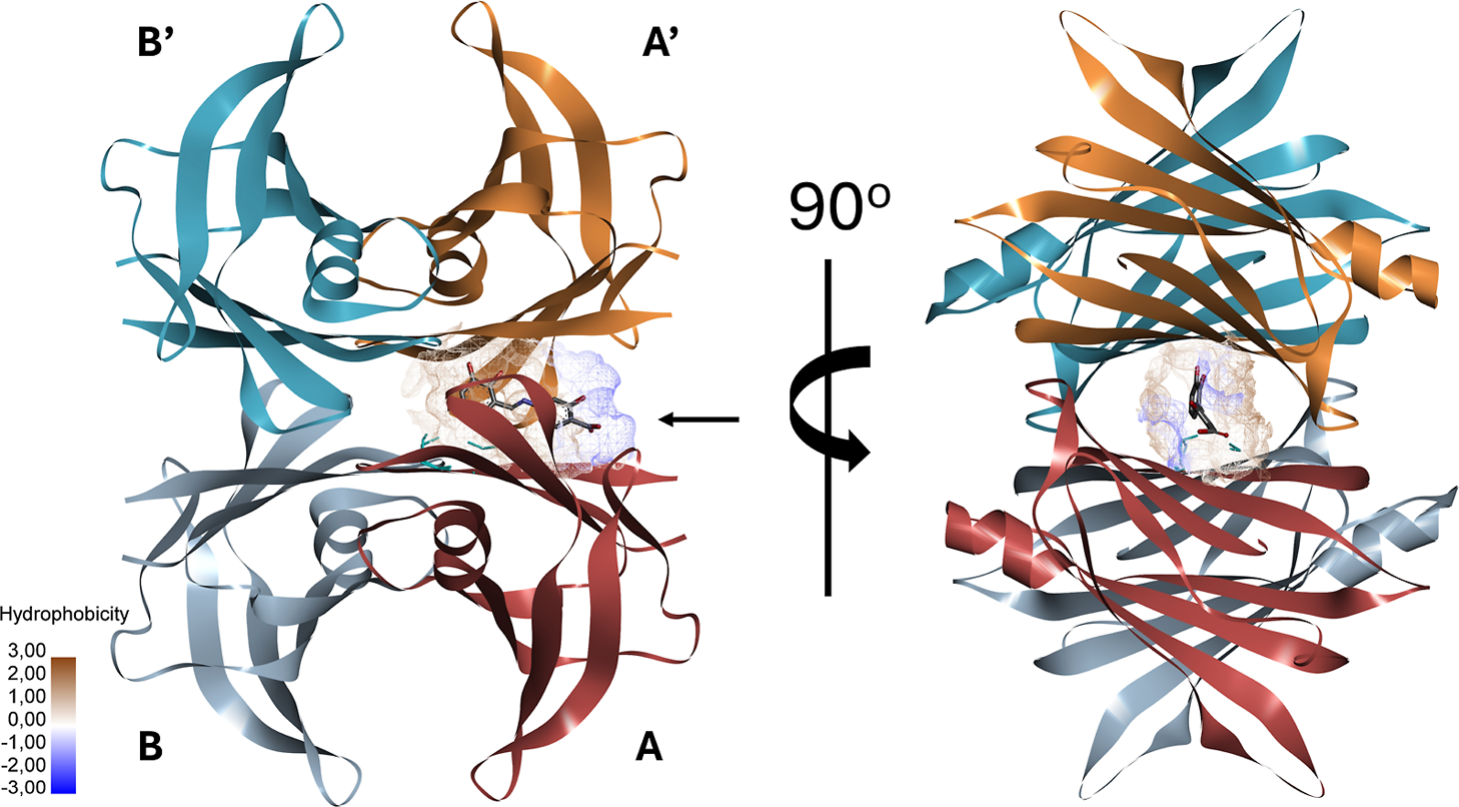
Human transthyretin tetramer structure in complex with 4-{(*E*)-[(2,3-dihydroxyphenyl)­methylidene]­amino}-2-hydroxybenzoic acid (stick model) bound in the central hydrophobic channel in the most stable pose. The four monomers are labeled A, B, A′, and B′. Subunits A–B and A′–B′ form two symmetric dimers that assemble into the functional tetramer.

**Table 4:** Summary of Binding Affinity, Ligand Efficiency, and Key Interactions of Tolcapone and the Obtained Compound with TTR^a^

compound (pose)	binding affinity (kJ/mol)	ligand efficiency	H-bonded amino acid residues	π-kind interactions with amino acid residues
Tolcapone (experimental)	-	-	Lys15	Ala108, Leu17
				Leu110, Lys15
Tolcapone (redocked)	–8.0	–0.40	Lys15	Ala108
				Leu110, Lys15
obtained compound (best pose)	–8.6	–0.43	Lys15	Ala108, Leu17
			Ser117	Leu110, Lys15

a van der Waals interactions were not taken into account in the analysis.

In accordance with the criteria proposed by Yokoyama et al.,^119^ a promising TTR stabilizer is expected to meet the following thresholds: binding affinity ≥−7.8 kcal/mol, ligand efficiency ≤−0.45, and structural similarity to known TTR stabilizers, requiring a Tanimoto coefficient of ≥0.3 with at least two reference compounds. Molecular docking simulations for 4-{(*E*)-[(2,3-dihydroxyphenyl)­methylidene]­amino}-2-hydroxybenzoic acid revealed that this molecule binds strongly (binding affinity of −8.6 kcal/mol) within the crystal structure of biologically active wild-type human transthyretin (TTR). Regarding the second criterion, the compound exhibited a ligand efficiency of −0.43, which is close to the required threshold and comparable to that of tolcapone (−0.40). The third criterion was also satisfied: as shown in Table 4, the compound achieved a Tanimoto coefficient of 0.3 with both diflunisal and tolcapone. Additionally, considering that the molecule contains two aromatic rings and an acidic functional group, both identified as important structural features for TTR stabilization, we conclude that the obtained compound represents a promising TTR stabilizer candidate. Notably, both the obtained compound and tolcapone appear to be primarily stabilized by hydrophobic and electrostatic interactions, which as reported by Petrassi et al.^120^ and Hammarström et al.^121^ play a key role in maintaining tetramer integrity. The docking results are presented in Tables S6 and S7 in the Supporting Information.

Within the TTR binding cavity, several key amino acid residues—Lys15, Leu17, Ala108, Thr119, Leu110, and Ser117—define three distinct binding pockets, designated as P1, P2, and P3.^123^ It is predicted that the obtained compound can interact with key amino acid residues through strong hydrogen bonds (Lys15, Ser117), π-type interactions (Ala108, Leu17, Leu110, and Lys15), and van der Waals forces, as shown in Figure 14. These interactions are consistent with previously characterized binding patterns of polyphenol-based ligands such as quercetin, apigenin, and pterostilbene.^113^ As stated, a promising TTR stabilizer is expected to form interactions across all three binding pockets and preferentially engage two sites simultaneously to enhance occupancy and promote tetramer stabilization.^124^ The obtained compound fulfills this criterion as it is predicted to form hydrogen bonds with Lys15 (P1) and Ser117 (P3), as well as hydrophobic interactions with Ala108, Leu17, and Leu110 (P2). The potential binding to these residues may therefore contribute to TTR tetramer stabilization, similar to the effects observed for thyroxine, resveratrol, Tafamidis, and curcumin.^125–127^

**Figure 14:**
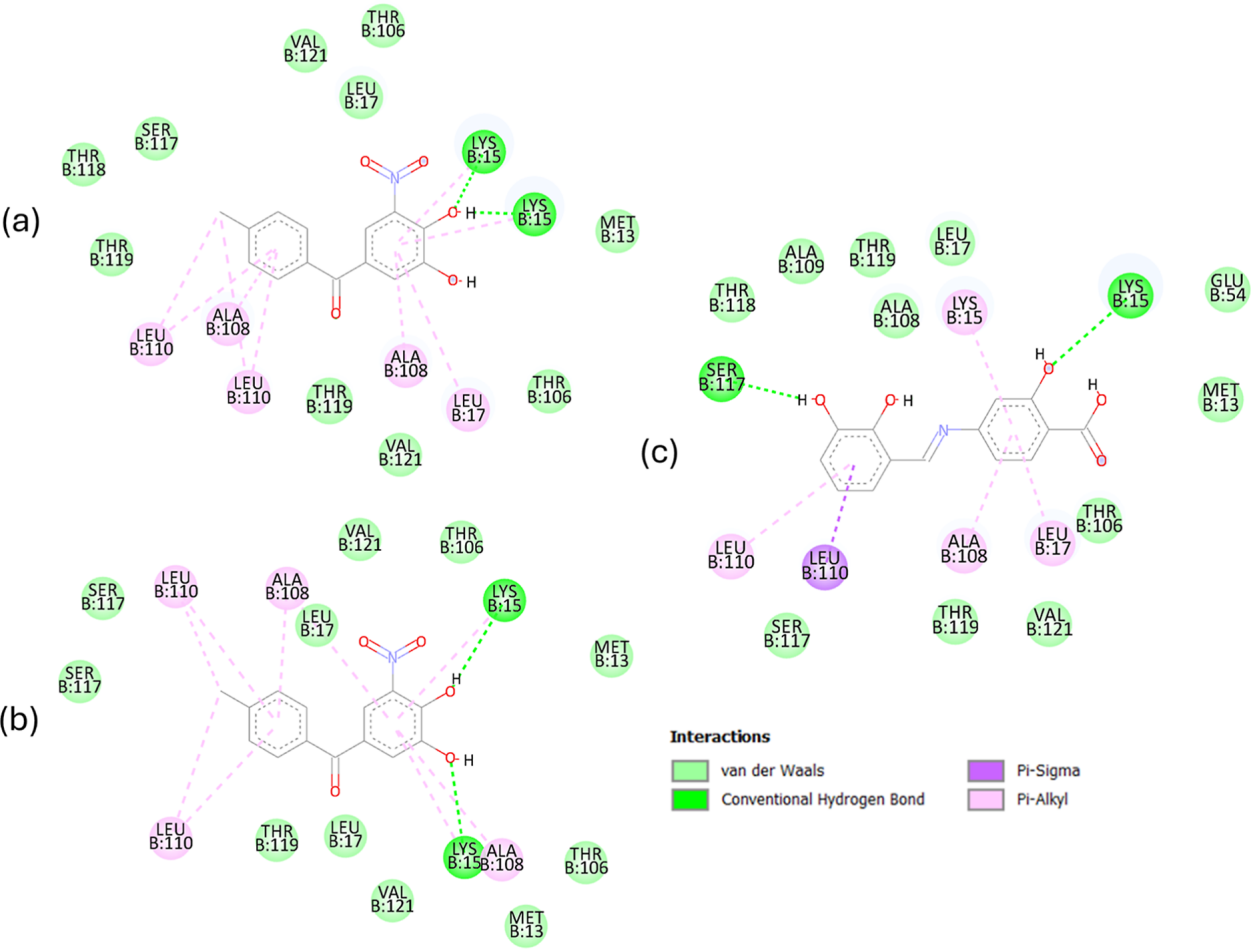
2D diagrams of interactions with amino acid residues for (a) experimental pose of Tolcapone, (b) redocking pose of Tolcapone, and (c) the best pose of the obtained compound docked in the TTR active site.

## Conclusions

4-{(*E*)-[(2,3-Dihydroxyphenyl)­methylidene]­amino}-2-hydroxybenzoic acid was synthesized and characterized via structural, spectroscopic, thermal, and computational methods. The ATR-FTIR spectrum supports Schiff base formation, as evidenced by the disappearance of bands characteristic of the primary amine and aldehyde functional groups and the appearance of a distinct azomethine (−HC=N−) stretching vibration at 1622 cm^–1^. The carboxylic C=O stretching band is shifted due to strong hydrogen bonding, indicating involvement of the carboxyl group in intermolecular interactions. Thermal analysis reveals a three-step decomposition process beginning with melting, confirming good thermal stability up to the onset of decomposition. Crystallographic study shows that the compound crystallizes in the triclinic *P̅*1 space group with two molecules in the asymmetric unit, both adopting the keto form in a zwitterionic imine state due to intramolecular proton transfer. This is further supported by shortened C–O and C–C bond lengths and a C–N bond typical of the keto tautomer, consistent with data from related phenol–imine compounds. Interaction energy calculations confirm the dominance of the zigzag chain motif. The π–π stacking interactions between conformer molecules highlight the role of dispersion forces in the packing arrangement. Frontier molecular orbital analysis reveals a HOMO–LUMO gap of 2.341 eV, indicating a low chemical reactivity and high kinetic stability. Localization of HOMO and LUMO on distinct molecules suggests intermolecular charge transfer via hydrogen-bonded heterosynthons, particularly involving N_(imine)_–H···O_(carboxyl)_ and O_(carboxyl)_–H···O_(hydroxyl)_ interactions. ADME and toxicity predictions suggest high gastrointestinal absorption, low acute toxicity, and potential transthyretin inhibitory activity. The compound exhibits favorable binding affinity and interaction profiles comparable to those of Tolcapone, highlighting its strong potential as a novel TTR stabilizer and positioning it as a promising scaffold for the rational design of next-generation therapeutics.

## Supplementary Material





## Notes

Dark-red crystals of 4-{(E)-[(2,3-dihydroxyphenyl)­methylidene]­amino}-2-hydroxybenzoic acid, mechanism of synthesis of 4-{(E)-[(2,3-dihydroxyphenyl)­methylidene]­amino}-2-hydroxybenzoic acid, melting point results for the crystal samples of 4-{(E)-[(2,3-dihydroxyphenyl)­methylidene]­amino}-2-hydroxybenzoic acid, energy framework data, ADMET data, ProTox results, hydrogen bond geometry for 4-{(E)-[(2,3 dihydroxyphenyl)­methylidene]­amino}-2-hydroxybenzoic acid, π–π stacking interaction geometry, C–O···π interaction geometry, molecular docking results for TTR binding, and molecular redocking results for TTR binding with tolcapone (PDF)
Crystallographic data (CIF)
