# Supplementary material for: Synthesis, Structural Characterization, and In Silico Evaluation of the Salicylidene Schiff Base 4‑{(E)‑[(2,3-Dihydroxyphenyl)methylidene]amino}-2-hydroxybenzoic Acid as a Promising Scaffold for Human Transthyretin Inhibitor
Source: ACS Omega. 2026 Jan 21;11(4):5154–69. doi: 10.1021/acsomega.5c07247 (PMC12878484; doi:10.1021/acsomega.5c07247)
Supplement: Supplementary file 1 [file ao5c07247_si_001.pdf]

Supporting information

Synthesis, Structural Characterization, and In Silico Evaluation of the Salicylidene Schiff Base 4-{(E)-[(2,3-Dihydroxyphenyl)methylidene]amino}-2 hydroxybenzoic Acid as a Promising Scaffold for Human Transthyretin Inhibitor

Patryk Nowak* & Artur Sikorski*

University of Gdańsk, Faculty of Chemistry, W. Stwosza 63, 80-308 Gdańsk, Poland

*E-mail: patryk.nowak@phdstud.ug.edu.pl (P.N.); artur.sikorski@ug.edu.pl (A.S.)

Synthesis

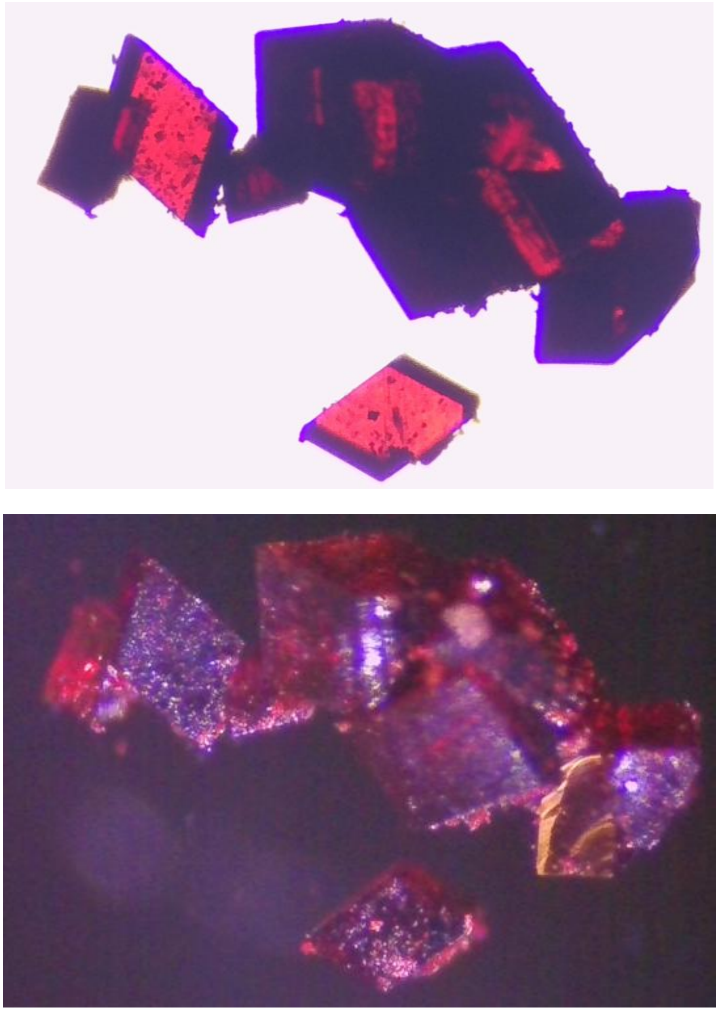

Figure S1. Dark red crystals of 4-{(E)-[(2,3-dihydroxyphenyl) methylidene]amino}-2-hydroxybenzoic acid.

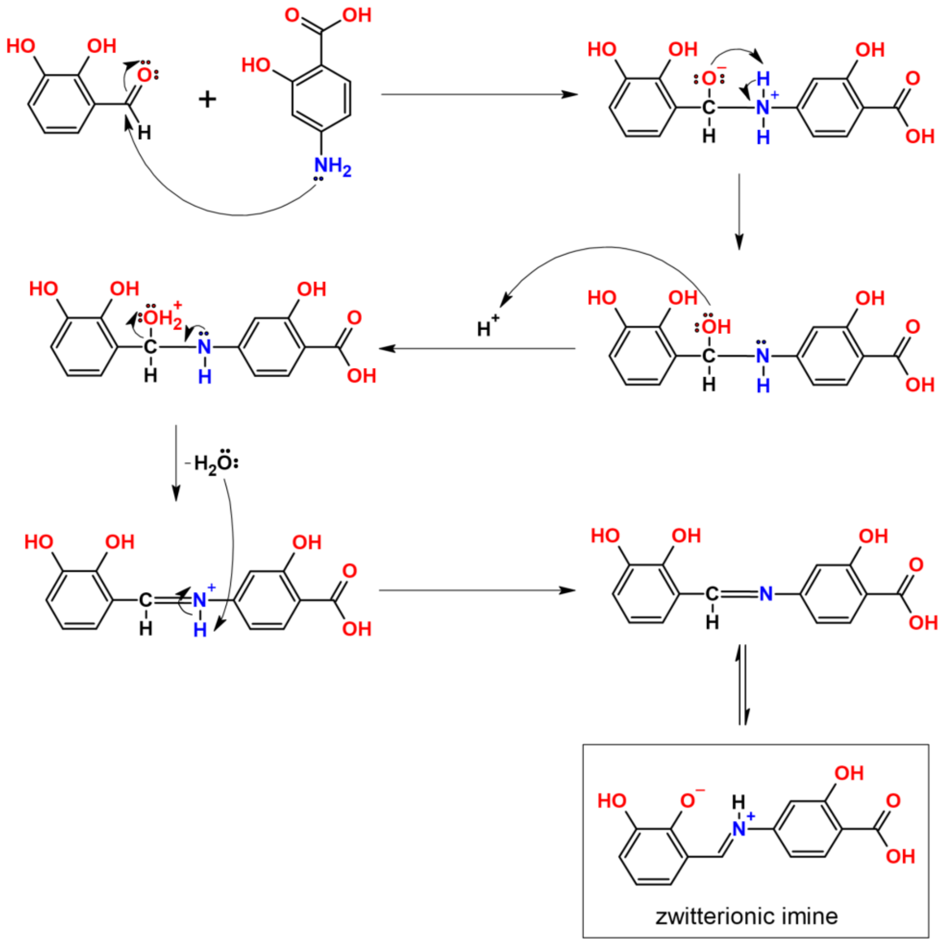

Figure S2. Mechanism of synthesis of 4-{(E)-[(2,3-dihydroxyphenyl)methylidene]amino}-2-hydroxybenzoic acid.

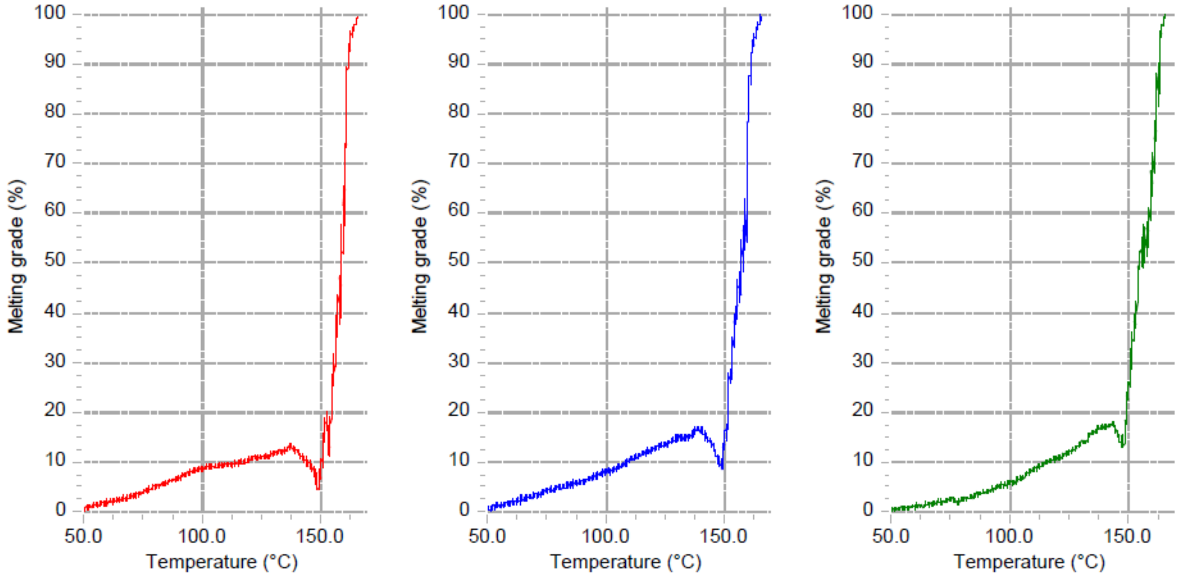

Figure S3. Melting point results for the crystal samples of 4-{(E)-[(2,3-dihydroxyphenyl)methylidene]amino}-2-hydroxybenzoic acid.

Energy framework data

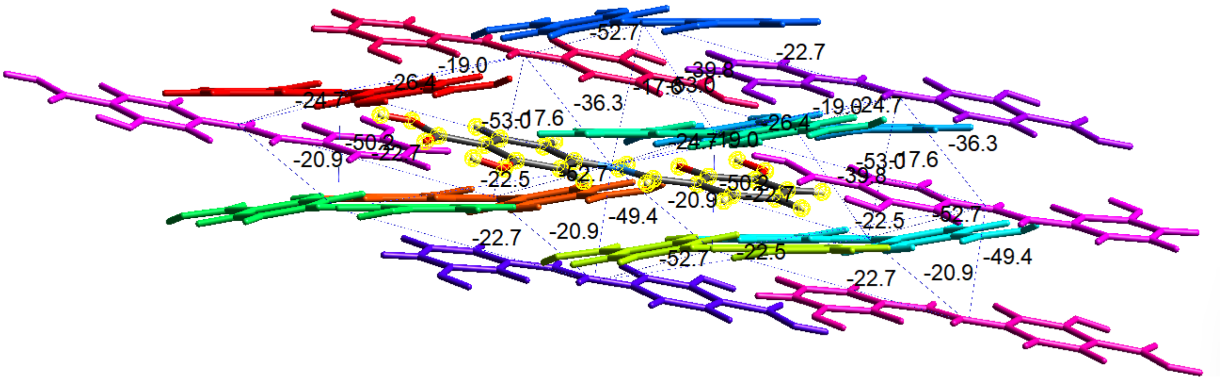

	N	Symop	R	Electron Density	E_ele	E_pol	E_dis	E_rep	E_tot
	1	-	9.15	B3LYP/6-31G(d,p)	-75.4	-17.8	-19.5	92.0	-53.0
	1	-	9.19	B3LYP/6-31G(d,p)	-68.5	-16.1	-19.6	78.8	-52.7
	0	-x, -y, -z	6.43	B3LYP/6-31G(d,p)	-7.2	-2.9	-66.0	44.5	-39.8
	0	-x, -y, -z	3.50	B3LYP/6-31G(d,p)	-2.0	-2.9	-87.6	49.2	-50.2
	1	-	9.21	B3LYP/6-31G(d,p)	-12.6	-2.5	-15.4	12.4	-20.9
	0	x, y, z	11.93	B3LYP/6-31G(d,p)	1.0	-0.3	-7.0	2.8	-3.5
	0	-x, -y, -z	12.65	B3LYP/6-31G(d,p)	-1.5	-0.2	-6.7	0.0	-7.6
	1	-	9.19	B3LYP/6-31G(d,p)	-14.1	-2.5	-16.5	14.0	-22.5
	1	-	9.47	B3LYP/6-31G(d,p)	-17.4	-3.5	-21.5	24.3	-24.7
	1	-	9.45	B3LYP/6-31G(d,p)	-12.2	-2.8	-19.1	14.6	-22.7
	1	-	10.93	B3LYP/6-31G(d,p)	-21.3	-4.1	-7.9	21.8	-19.0
	1	-	5.74	B3LYP/6-31G(d,p)	0.2	-0.9	-25.3	7.9	-17.6
	0	-x, -y, -z	8.86	B3LYP/6-31G(d,p)	0.2	-0.6	-4.6	0.4	-4.0
	1	-x, -y, -z	3.53	B3LYP/6-31G(d,p)	-3.0	-2.9	-89.1	54.2	-49.4
	1	-x, -y, -z	9.24	B3LYP/6-31G(d,p)	-6.2	-1.9	-39.2	25.2	-26.4
	2	x, y, z	11.93	B3LYP/6-31G(d,p)	1.8	-0.3	-6.2	2.1	-2.4
	1	-x, -y, -z	12.65	B3LYP/6-31G(d,p)	-1.8	-0.2	-5.8	0.0	-7.0
	1	-x, -y, -z	6.14	B3LYP/6-31G(d,p)	-25.7	-4.2	-30.8	33.6	-36.3

(a)

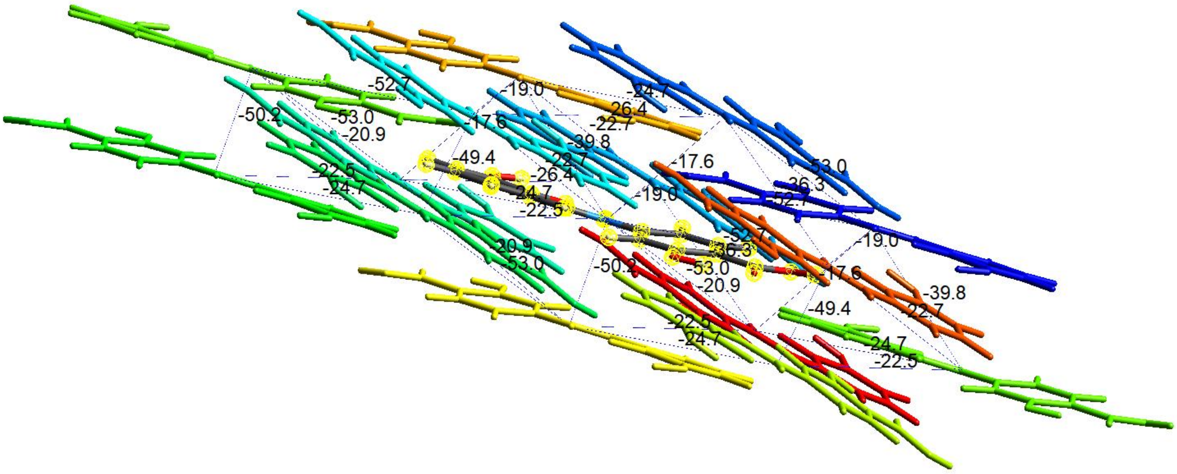

	N	Symop	R	Electron Density	E_ele	E_pol	E_dis	E_rep	E_tot
	1	-	9.15	B3LYP/6-31G(d,p)	-75.4	-17.8	-19.5	92.0	-53.0
	1	-	9.19	B3LYP/6-31G(d,p)	-68.5	-16.1	-19.6	78.8	-52.7
	1	-x, -y, -z	6.43	B3LYP/6-31G(d,p)	-7.2	-2.9	-66.0	44.5	-39.8
	1	-x, -y, -z	3.50	B3LYP/6-31G(d,p)	-2.0	-2.9	-87.6	49.2	-50.2
	1	-	9.21	B3LYP/6-31G(d,p)	-12.6	-2.5	-15.4	12.4	-20.9
	2	x, y, z	11.93	B3LYP/6-31G(d,p)	1.0	-0.3	-7.0	2.8	-3.5
	1	-x, -y, -z	12.65	B3LYP/6-31G(d,p)	-1.5	-0.2	-6.7	0.0	-7.6
	1	-	9.19	B3LYP/6-31G(d,p)	-14.1	-2.5	-16.5	14.0	-22.5
	1	-	9.47	B3LYP/6-31G(d,p)	-17.4	-3.5	-21.5	24.3	-24.7
	1	-	9.45	B3LYP/6-31G(d,p)	-12.2	-2.8	-19.1	14.6	-22.7
	1	-	10.93	B3LYP/6-31G(d,p)	-21.3	-4.1	-7.9	21.8	-19.0
	1	-	5.74	B3LYP/6-31G(d,p)	0.2	-0.9	-25.3	7.9	-17.6
	1	-x, -y, -z	8.86	B3LYP/6-31G(d,p)	0.2	-0.6	-4.6	0.4	-4.0
	0	-x, -y, -z	3.53	B3LYP/6-31G(d,p)	-3.0	-2.9	-89.1	54.2	-49.4
	0	-x, -y, -z	9.24	B3LYP/6-31G(d,p)	-6.2	-1.9	-39.2	25.2	-26.4
	0	x, y, z	11.93	B3LYP/6-31G(d,p)	1.8	-0.3	-6.2	2.1	-2.4
	0	-x, -y, -z	12.65	B3LYP/6-31G(d,p)	-1.8	-0.2	-5.8	0.0	-7.0
	0	-x, -y, -z	6.14	B3LYP/6-31G(d,p)	-25.7	-4.2	-30.8	33.6	-36.3

(b)

Figure S4. Energy framework results for 4-{(E)-[(2,3-dihydroxyphenyl)methylidene]amino}-2-hydroxybenzoic acid, shown for (a) conformer 1 and (b) conformer 2, with neighboring molecules generated within a 3.8 Å radius.

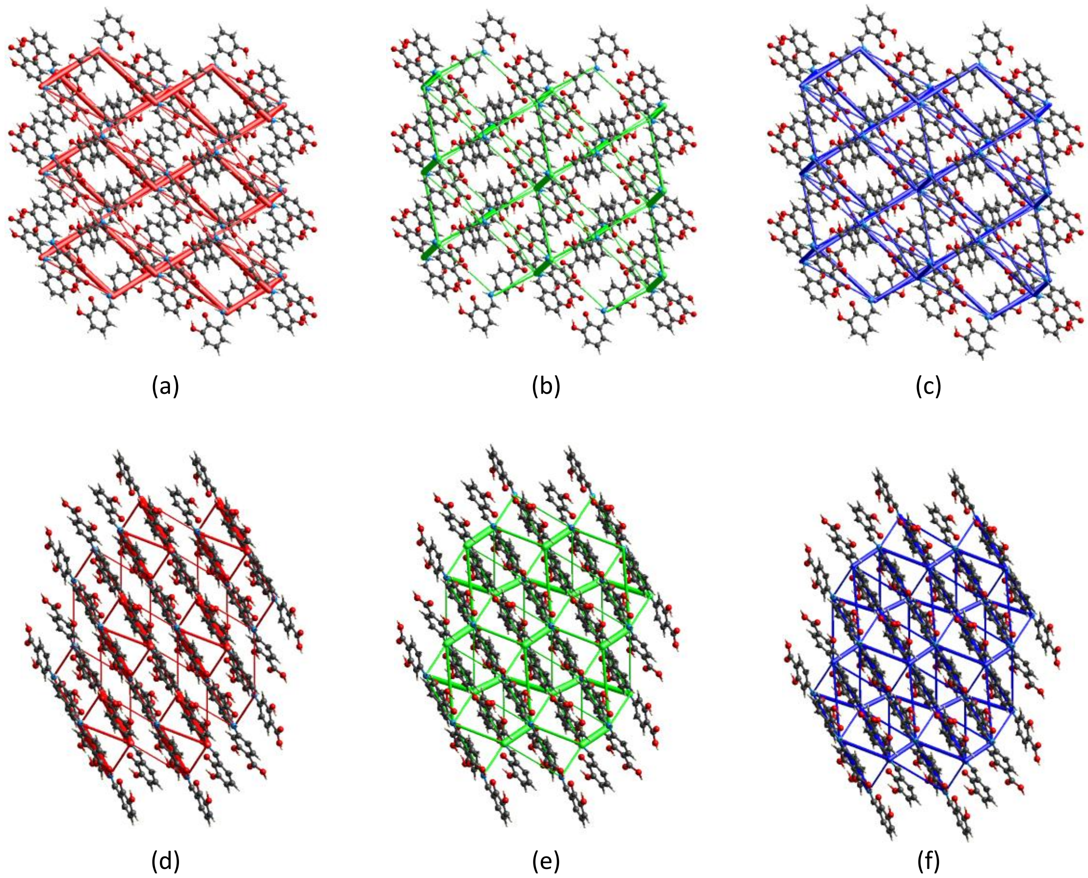

Figure S5. Energy frameworks for a molecular cluster of the 4-{(E)-[(2,3-dihydroxyphenyl)methylidene]amino}-2-hydroxybenzoic acid crystal, depicting electrostatic energy, dispersion energy, and total energy viewed along *a*-axis (a-c, respectively) and *c*-axis (d-f, respectively). The tube size is set to 50, with a cut-off of 10 kJ/mol.

ADMET data

Table S1. ADME results for 4-{(E)-[(2,3-dihydroxyphenyl)methylidene]amino}-2-hydroxybenzoic acid**.**

**Physicochemical properties**	
Molecular formula	$C_{14}H_{11}NO_5$
Molecular Weight (g/mol)	273.24
Number of heavy atoms	20
Number of aromatic heavy atoms	12
Number of rotating bonds	3
Number of hydrogen bond acceptors	6
Number of hydrogen bond donors	4
Molar refracdance	73.17
Topological polar surface (TPSA) (Å²)	110.35
**Lipophilicity**	
Log $P_{o/w}$ (iLOGP)	2.02
Log $P_{o/w}$ (XLOGP3)	2.23
Log $P_{o/w}$ (WLOGP)	2.25
Log $P_{o/w}$ (MLOGP)	1.03
Log $P_{o/w}$ (SILICOS-IT)	1.81
Consensus Log $P_{o/w}$	1.87
**Water solubility**	
Log S (ESOL)	-3.18
Solubility (mg/ml)	0.178
Class	Soluble
Log S (Ali)	-4.18
Solubility (mg/ml)	0.018
Class	Soluble
Log S (SILICOS-IT)	-2.66
Solubility (mg/ml)	0.603
Class	Soluble
**Pharmacokinetics**	
Absorption GI	High
Permeability BBB	No
Substrat P-gp	No
**inhibitor CYP1A2**	**Yes**
inhibitor CYP2C19	No
inhibitor CYP2C9	No
inhibitor CYP2D6	No
**inhibitor CYP3A4**	**Yes**
Log K_p (penetration through the skin)	-6.38 cm/s
**Similarity to drug**	
Lipinski criteria (Pfizer)	Yes
Ghose criteria	Yes
Veber criteria (GSK)	Yes
Egan criteria (Pharmacia)	Yes
Muegge criteria (Bayer)	Yes
Bioavailability index	0.56
**Other**	
PAINS (Interference structures)	1 alert: katechol
Brenk (Structural alerts)	2 alerts: katechol, imine
Similarity to the leading compound	Yes
Easy to synthesize	2.57

Table S2. ProTox results for 4-{(E)-[(2,3-dihydroxyphenyl)methylidene]amino}-2-hydroxybenzoic acid.

Classification	Target	Prediction	Probability
Organ toxicity	Hepatotoxicity	Active	0.64
Organ toxicity	Neurotoxicity	Inactive	0.66
Organ toxicity	Nephrotoxicity	Active	0.70
Organ toxicity	Respiratory toxicity	Active	0.72
Organ toxicity	Cardiotoxicity	Inactive	0.63
Toxicity end points	Carcinogenicity	Active	0.51
Toxicity end points	Immunotoxicity	Inactive	0.98
Toxicity end points	Mutagenicity	Inactive	0.68
Toxicity end points	Cytotoxicity	Active	0.52
Toxicity end points	BBB-barrier	Inactive	0.68
Toxicity end points	Ecotoxicity	Inactive	0.60
Toxicity end points	Clinical toxicity	Active	0.59
Toxicity end points	Nutritional toxicity	Inactive	0.73
Tox21-Nuclear receptor signalling pathways	Aryl hydrocarbon Receptor (AhR)	Inactive	0.56
Tox21-Nuclear receptor signalling pathways	Androgen Receptor (AR)	Inactive	0.96
Tox21-Nuclear receptor signalling pathways	Androgen Receptor Ligand Binding Domain (AR-LBD)	Inactive	0.99
Tox21-Nuclear receptor signalling pathways	Aromatase	Inactive	0.81
Tox21-Nuclear receptor signalling pathways	Estrogen Receptor Alpha (ER)	Active	0.53
Tox21-Nuclear receptor signalling pathways	Estrogen Receptor Ligand Binding Domain (ER-LBD)	Inactive	0.60
Tox21-Nuclear receptor signalling pathways	Peroxisome Proliferator Activated Receptor Gamma (PPAR-Gamma)	Inactive	0.97
Tox21-Stress response pathways	Nuclear factor (erythroid-derived 2)-like 2/antioxidant responsive element (nrf2/ARE)	Inactive	0.79
Tox21-Stress response pathways	Heat shock factor response element (HSE)	Inactive	0.79
Tox21-Stress response pathways	Mitochondrial Membrane Potential (MMP)	Inactive	0.50
Tox21-Stress response pathways	Phosphoprotein (Tumor Supressor) p53	Inactive	0.77
Tox21-Stress response pathways	ATPase family AAA domain-containing protein 5 (ATAD5)	Inactive	0.60
Molecular Initiating Events	Thyroid hormone receptor alpha (THRα)	Active	0.54
Molecular Initiating Events	Thyroid hormone receptor beta (THRβ)	Inactive	0.87
**Molecular Initiating Events**	**Transtyretrin (TTR)**	**Active**	**0.63**
Molecular Initiating Events	Ryanodine receptor (RYR)	Inactive	0.91
Molecular Initiating Events	GABA receptor (GABAR)	Inactive	0.83
Molecular Initiating Events	Glutamate N-methyl-D-aspartate receptor (NMDAR)	Inactive	0.95
Molecular Initiating Events	alpha-amino-3-hydroxy-5-methyl-4-isoxazolepropionate receptor (AMPAR)	Inactive	0.99
Molecular Initiating Events	Kainate receptor (KAR)	Inactive	0.99
Molecular Initiating Events	Achetylcholinesterase (AChE)	Inactive	0.95
Molecular Initiating Events	Constitutive androstane receptor (CAR)	Inactive	1
Molecular Initiating Events	Pregnane X receptor (PXR)	Inactive	0.74
Molecular Initiating Events	NADH-quinone oxidoreductase (NADHOX)	Inactive	0.89
Molecular Initiating Events	Voltage gated sodium channel (VGSC)	Inactive	0.83
Molecular Initiating Events	Na+/I- symporter (NIS)	Inactive	0.95
Metabolism	Cytochrome CYP1A2	Inactive	0.58

Metabolism	Cytochrome CYP2C19	Inactive	0.79
Metabolism	Cytochrome CYP2C9	Active	0.52
Metabolism	Cytochrome CYP2D6	Inactive	0.79
Metabolism	Cytochrome CYP3A4	Inactive	0.83
Metabolism	Cytochrome CYP2E1	Inactive	0.99

Crystallographic data

Table S3. Hydrogen bonds geometry for 4-{(E)-[(2,3-dihydroxyphenyl)methylidene]amino}-2-hydroxybenzoic acid.

D–H···A	*d*(D–H) [Å]	*d*(H···A) [Å]	*d*(D···A) [Å]	∠D–H···A (°)
O9–H9···O38^{i}	0.88(4)	1.72(4)	2.56(3)	160(4)
O19–H19···O9^{ii}	0.81(4)	2.43(4)	3.05(3)	134(4)
O29–H29···O18^{iii}	0.98(4)	1.67(4)	2.636(3)	169(4)
O39–H39···O19^{ii}	0.77(4)	2.18(4)	2.868(3)	150(4)'
C3–H3···O28^{iv}	0.93	2.58	3.416(3)	149
C5–H5···O28^{v}	0.93	2.57	3.224(3)	128
C6–H6···O18^{iv}	0.93	2.60	3.357(3)	139
C11–H11···O39^{vi}	0.93	2.51	3.186(3)	130
C23–H23···O8^{vii}	0.93	2.58	3.431(3)	153

Symmetry code: (i) x, y, z; (ii) 1-x, 2-y, 2-z; (iii) x, y, -1+z; (iv) -x, 2-y, 1-z; (v) x, y, 1+z; (vi) -1+x, 1+y, z; (vii) 1-x, 1-y, 1-z.

Table S4. π–π stacking interactions geometry for 4-{(E)-[(2.3-dihydroxyphenyl)methylidene]amino}-2-hydroxybenzoic acid.

CgI[a]	CgJ[a]	CgI···CgJ[b] [Å]	Dihedral angle[c] [°]	Interplanar distance[d] [Å]	Offset[e] [Å]
Cg(1)	Cg(2)^{i}	3.719(13)	6.25(11)	3.3312(10)	1.356
Cg(2)	Cg(2)^{ii}	3.463(14)	0.00(12)	3.4310(10)	0.467
Cg(3)	Cg(4)^{iii}	3.709(15)	10.32(12)	3.4420(10)	1.266

Symmetry code: (i) -x, 2-y, 2-z; (ii) -x, 3-y, 2-z; (iii) 1-x, 1-y, 1-z.

(a) Cg represents the centre of gravity of the rings. (b) Cg···Cg is the distance between ring centroids, (c) The dihedral angle is that between the mean planes of Cg(I) on ring J, (d) The interplanar distance is the perpendicular distance from CgI to ring J, (e) The offset is the perpendicular distance of CgJ on ring I.

Table S5. C–O···π interaction geometry for 4-{(E)-[(2.3-dihydroxyphenyl)methylidene]amino}-2-hydroxybenzoic acid.

C–O···Cg(I)	*d*(O···Cg(I)) [Å]	*d*(C···Cg(I)) [Å]	∠C–O···Cg(I) [°]
C33–O38···Cg4^{i}	3.600	3.979	97.6(13)

Symmetry code: (i) 2-x, 1-y, 1-z.

Molecular docking

Table S6. Molecular docking results for TTR binding with 4-{(E)-[(2,3-dihydroxyphenyl)methylidene]amino}-2-hydroxybenzoic acid.

Mode	Affinity (kcal/mol)	Distance from best mode	
		R.M.S.D l.b	R.M.S.D u.b
1	-8.6	0.000	0.000
2	-8.6	2.527	3.713
3	-8.2	1.531	2.000
4	-7.8	2.814	7.568
5	-7.8	2.406	7.868
6	-7.7	1.638	7.945
7	-7.6	1.638	7.854
8	-7.2	2.384	3.633
9	-7.2	3.267	4.554
10	-7.1	2.406	1.972

Table S7. Molecular redocking results for TTR binding with Tolcapone.

Mode	Affinity (kcal/mol)	Distance from best mode	
		R.M.S.D l.b	R.M.S.D u.b
1	-9.2	0.000	0.000
2	-8.9	2.178	2.628
3	-8.0	3.340	6.372
4	-8.0	3.451	6.836
5	-7.4	2.991	5.306
6	-7.4	4.358	5.720
7	-7.0	8.477	11.600
8	-6.8	8.969	11.612
9	-6.8	8.381	11.694
10	-6.7	8.413	11.785
